# Viral delivery of an RNA-guided genome editor for transgene-free germline editing in *Arabidopsis*

**DOI:** 10.1038/s41477-025-01989-9

**Published:** 2025-04-22

**Authors:** Trevor Weiss, Maris Kamalu, Honglue Shi, Zheng Li, Jasmine Amerasekera, Zhenhui Zhong, Benjamin A. Adler, Michelle M. Song, Kamakshi Vohra, Gabriel Wirnowski, Sidharth Chitkara, Charlie Ambrose, Noah Steinmetz, Ananya Sridharan, Diego Sahagun, Jillian F. Banfield, Jennifer A. Doudna, Steven E. Jacobsen

**Affiliations:** 1 Department of Molecular, Cell and Developmental Biology, University of California at Los Angeles, Los Angeles, CA USA; 2 Innovative Genomics Institute, University of California, Berkeley, CA USA; 3 Howard Hughes Medical Institute, University of California, Berkeley, CA USA; 4 California Institute for Quantitative Biosciences (QB3), University of California, Berkeley, CA USA; 5 Department of Earth and Planetary Science, University of California, Berkeley, CA USA; 6 Department of Environmental Science, Policy and Management, University of California, Berkeley, CA USA; 7 University of Melbourne, Melbourne, Australia; 8 Department of Molecular and Cell Biology, University of California, Berkeley, CA USA; 9 Department of Chemistry, University of California, Berkeley, CA USA; 10 Molecular Biophysics and Integrated Bioimaging Division, Lawrence Berkeley National Laboratory, Berkeley, CA USA; 11 Li Ka Shing Center for Translational Genomics, University of California, Berkeley, CA USA; 12 Gladstone Institute of Data Science and Biotechnology, San Francisco, CA USA; 13 Gladstone-UCSF Institute of Genomic Immunology, San Francisco, CA USA; 14 Howard Hughes Medical Institute (HHMI), University of California at Los Angeles, Los Angeles, CA USA; 15 Present Address: Ministry of Education Key Laboratory for Bio-Resource and Eco-Environment, College of Life Sciences, State Key Laboratory of Hydraulics and Mountain River Engineering, Sichuan University, Chengdu, China

**Keywords:** Molecular engineering in plants, Genetic engineering

## Abstract

Genome editing is transforming plant biology by enabling precise DNA modifications. However, delivery of editing systems into plants remains challenging, often requiring slow, genotype-specific methods such as tissue culture or transformation^1^. Plant viruses, which naturally infect and spread to most tissues, present a promising delivery system for editing reagents. However, many viruses have limited cargo capacities, restricting their ability to carry large CRISPR-Cas systems. Here we engineered tobacco rattle virus (TRV) to carry the compact RNA-guided TnpB enzyme ISYmu1 and its guide RNA. This innovation allowed transgene-free editing of *Arabidopsis thaliana* in a single step, with edits inherited in the subsequent generation. By overcoming traditional reagent delivery barriers, this approach offers a novel platform for genome editing, which can greatly accelerate plant biotechnology and basic research.

## Abstract (web summary)

Weiss et al. engineered the tobacco rattle virus to express the RNA-guided TnpB enzyme ISYmu1 and guide RNA for transgene-free germline editing in *Arabidopsis thaliana*.

## Main

Programmable RNA-guided endonucleases, including CRISPR-Cas9, are driving advances in genome editing for both fundamental research and biotechnology. The ability to genetically modify plant genomes has allowed for the creation of rationally designed phenotypes. However, efficient delivery of genome editing reagents to plants remains a major challenge. The most common strategy is to encode RNA-guided genome editors (for example, CRISPR-Cas enzymes) within transgenes and use tissue culture and plant transformation approaches to make transgenic plants, after which genetic crosses are required to remove the transgenic material but retain the edits^1–3^. However, current plant transformation methods are limited to specific plant species and genotypes, often require considerable time, resources and technical expertise, and can cause unintended changes to the genome and epigenome^1^.

An approach to circumvent these limitations is to use plant viral vectors to deliver genome editing reagents such as meganucleases or zinc finger nucleases (ZFNs) for targeted mutagenesis^4,5^. While the use of meganucleases and ZFNs for viral-mediated plant genome editing was a notable advance, the ability to encode an easily programmable RNA-guided CRISPR system would be highly advantageous. As such, several viral vectors have been engineered to encode guide RNAs (gRNAs) for delivery to transgenic plants already expressing Cas9, resulting in somatic and germline editing and transmission of edits to the next generation^6–9^. Because plants have evolved mechanisms to restrict viral infection of meristem and germ cells, most viruses are rarely sexually transmitted^10^. However, transient invasion of meristem cells by viral RNAs encoding gRNAs can allow these cells to be edited and for these edits to be seed transmissible^6–9^. While these approaches represent important advances, they still require the use of nucleases that can be challenging to engineer (such as meganucleases and ZFNs), or transgenic plants expressing the CRISPR-Cas endonuclease protein.

A strategy to avoid the need for transgenic plant materials has been the use of viral vectors with large cargo capacities, capable of expressing entire RNA-guided editing systems (for example, Cas9 and the gRNA). This approach has been met with some success; however, it still requires plant regeneration steps because these viruses do not cause germline editing and heritability of the edits^11–14^. On the other hand, encoding entire CRISPR systems in viruses that are capable of germline transmission has been challenging because of their limited cargo capacity^6–9,15^.

To overcome this cargo size limit, we explored the potential of TnpB, a class of ultracompact RNA-guided endonucleases (~400 amino acids)^16–18^, to be encoded in a plant RNA viral vector. As ancestors of Cas enzymes, TnpBs similarly utilize a programmable RNA guide, called an omega RNA (ωRNA), to be directed to any target site and induce genome edits. Previously, TnpBs ISDra2, ISYmu1 and ISAam1 were shown to be capable of targeted genome editing in mammalian cells, and ISDra2 and ISYmu1 in monocot rice plant cells^16,19–21^. Here we tested the ISDra2, ISYmu1 and ISAam1 TnpBs for genome editing in the dicot plant, *Arabidopsis*. Given the single cargo site in the TRV vector that is typically used, we sought to express both the TnpB protein and its guide RNA within the same mRNA transcript under a single promoter, similar to their natural expression arrangement^16–18^.

**Fig. 1:**
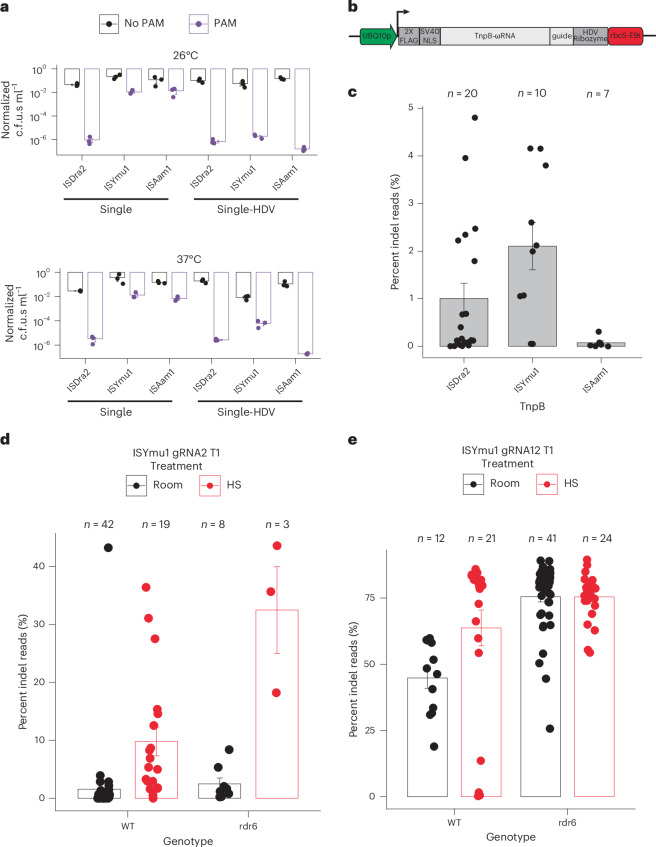
Expression of TnpB and guide RNA in a single transcript for plant genome editing. **a**, Barplots of interference assay testing the single transcript expression TnpB vectors for cleavage in *E. coli*. Data are from experiments performed at 26 °C (top) and at 37 °C (bottom). Bars indicate absence (black) or presence (purple) of a PAM on the target plasmid. The *Y* axis is a log_10_ scale of the normalized c.f.u.s ml^−1^. The *X* axis displays the three TnpBs tested using the single expression transcript design without or with an HDV ribozyme. The s.e.m. was calculated for each experiment, with 3 replicates per experiment. **b**, Schematic of the single expression transcript TnpB-ωRNA plasmid design used for plant genome editing. The green arrow symbolizes the *AtUBQ10* promoter; the dark grey boxes indicate the 2×-FLAG, SV40 NLS and HDV ribozyme sequences; the light grey boxes indicate the TnpB-ωRNA and guide sequences; the red box symbolizes the rbcS-E9 terminator; the black arrow indicates the orientation of the TnpB-ωRNA expression cassette. **c**, Barplot displaying the average editing efficiencies (±s.e.m.) for protoplast experiments using ISDra2, ISYmu1 and ISAam1 TnpBs. Each dot represents the average editing efficiency (percent indel reads) of a gRNA from Extended Data Fig. 3a, with number of samples indicated at the top of the plot. **d**,**e**, ISYmu1 somatic editing in T1 transgenic plants for ISYmu1 gRNA2 (**d**) and ISYmu1 gRNA12 (**e**). The genotypes are plotted along the *X* axis and the editing efficiencies (percent indel reads) (±s.e.m.) are plotted on the *Y* axis. Each dot indicates a single T1 transgenic plant. The room and HS treatments stand for room temperature and heat-shock plant growth conditions, respectively.

**Extended Data Fig. 1:**
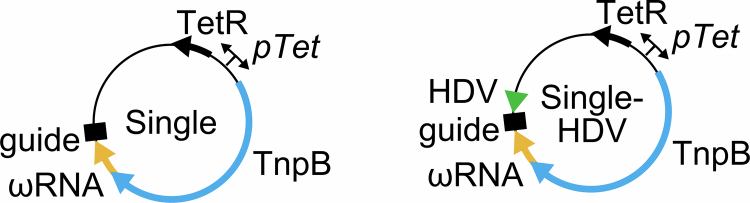
Schematic of bacterial interference assay plasmids containing either TnpB-ωRNA Single or TnpB-ωRNA Single-HDV design. The blue arrow indicates the TnpB sequence; the yellow arrow indicates the ωRNA sequence; the black rectangle indicates the guide sequence; the green arrow indicates the HDV ribozyme sequence. The plasmids contain the tetracycline resistance gene (TetR). A tetracycline promoter (pTet) was used to drive expression of the TnpB-ωRNA single or Single-HDV sequences, and the tetracycline resistance gene.

**Extended Data Fig. 2:**
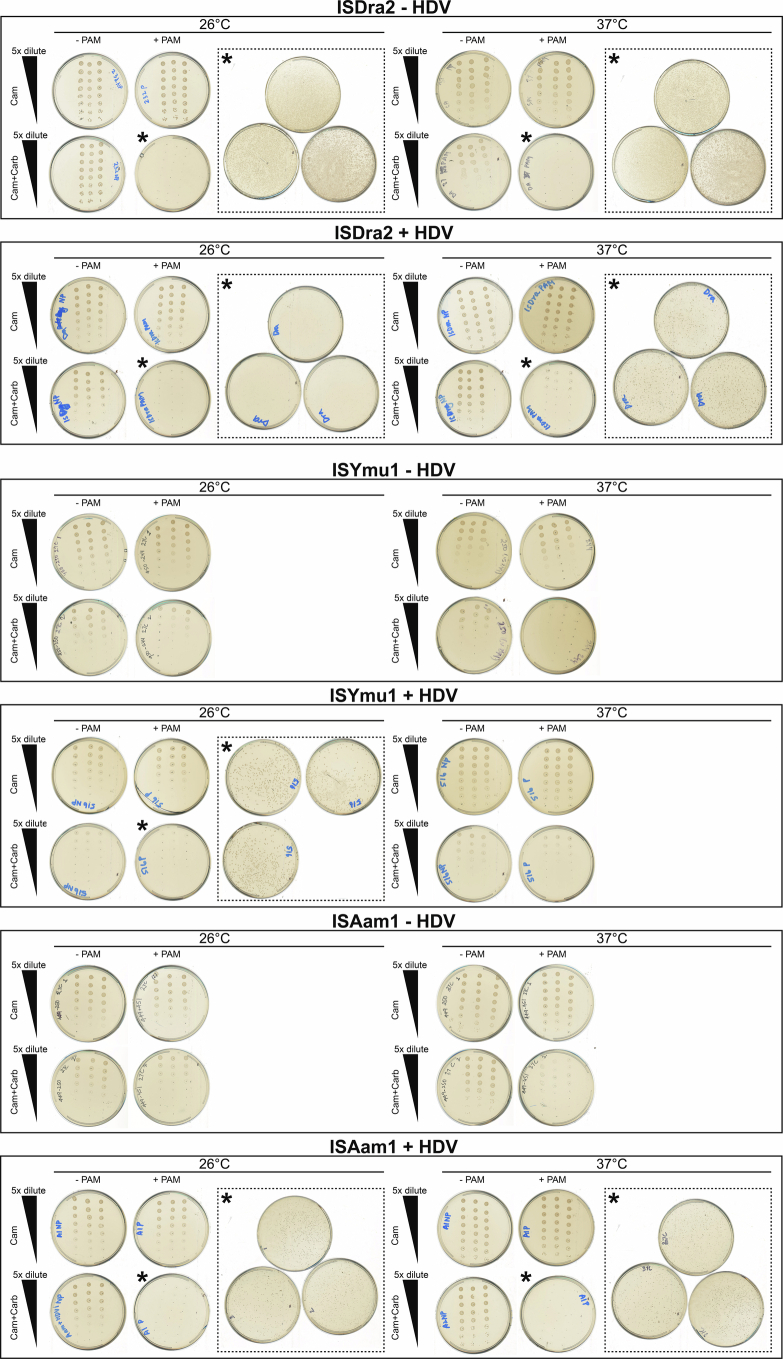
Plate images of bacterial plasmid interference assay. Five-fold serial dilutions (5 μL) from the 1 mL recovery culture post transformation were plated on both single antibiotic LB-Agar plates (Cam, upper row) and double antibiotic LB-Agar plates (Cam + Carb, lower row). Plates without visible colonies (indicated by an asterisk) had 400 μL of the original 1 mL recovery culture plated on the double antibiotic plates (dash insets). Experiments were performed in triplicates.

To test the activities of TnpB and its gRNA encoded in a single transcript, we first expressed these three TnpBs and assessed their RNA-guided plasmid interference activities in bacteria. We co-expressed the TnpB and gRNA from the same promoter as a single transcript, maintaining their natural sequences without codon optimization. We compared two configurations of the 3’-guide region: one extended continuously without a terminator to mimic the natural TnpB condition, and another capped by the hepatitis delta virus (HDV) ribozyme, as previously used in bacteria^16^ (Extended Data Fig. 1). Our results showed that without the HDV ribozyme, only ISDra2 demonstrated plasmid interference activity whereas with the HDV ribozyme, all three TnpBs exhibited robust activity at both 26 °C and 37 °C (Fig. 1a and Extended Data Fig. 2). These findings revealed that single transcript expression cassettes with an HDV ribozyme sequence at the 3’ end are capable of cleaving plasmid DNA in bacteria.

**Extended Data Fig. 3:**
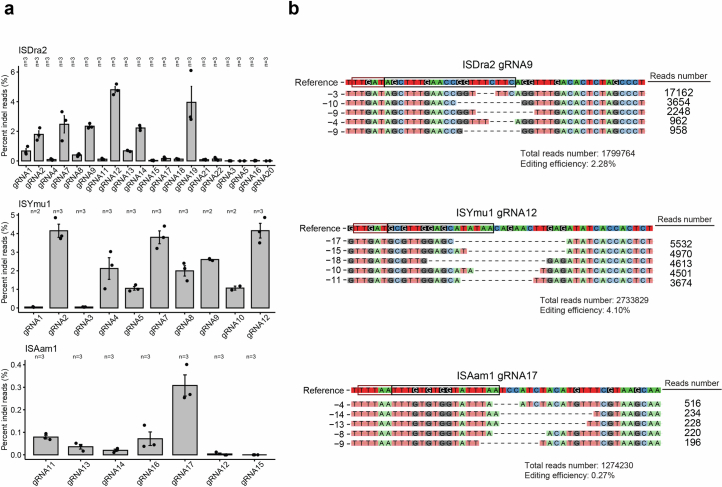
Editing efficiency and DNA repair profiles for ISDra2, ISYmu1, and ISAam1 in protoplast experiment. (**a**) The name of each TnpB tested is at the upper left of each bar plot. The gRNAs are plotted along the X-axis and the editing efficiency (percent indel reads (%)) is plotted on the Y-axis. Each dot indicates a single transfection. The standard error of the mean (SEM) was calculated for each target site. Two or three replicates were used for each target site tested. (**b**) DNA repair indel profiles for individual transfection samples. The top five most common indel types are listed on the left. The read counts for each indel are listed on the right. The PAM is identified by the red box, and the target site is outlined by the black box, in the Reference sequence. The total read number and editing efficiency are listed below each indel profile. The name of each TnpB and gRNA is displayed above the Reference sequence of each indel repair profile.

To test the single expression cassette for targeted genome editing in *Arabidopsis*, we used the *AtUBQ10* promoter to drive expression of the TnpB-ωRNA and a gRNA targeting the *PHYTOENE DESATURASE3* (*AtPDS3*) gene region, followed by the HDV ribozyme and rbcS-E9 terminator (Fig. 1b). We tested 20 ISDra2 sites, 10 ISYmu1 sites and 7 ISAam1 sites for editing capabilities in *Arabidopsis* protoplast cells (Supplementary Table 1)^22^. ISDra2 and ISYmu1 demonstrated active editing ranging from 0–4.8% and 0.1–4.2%, respectively, as measured by next-generation amplicon sequencing (amp-seq) (Extended Data Fig. 3a). ISAam1 was much less active, with editing efficiency ranging 0–0.3% (Extended Data Fig. 3a). On average, we observed editing efficiencies of 1% for ISDra2, 2.1% for ISYmu1 and 0.1% for ISAam1 (Fig. 1c). In line with previous reports, the DNA repair profiles consisted of deletion-dominant repair outcomes for all three TnpBs (Extended Data Fig. 3b)^16,19,20^. These data demonstrate that ISDra2, ISYmu1 and ISAam1 are all capable of targeted genome editing in *Arabidopsis* plant cells using the single transcript expression design.

**Extended Data Fig. 4:**
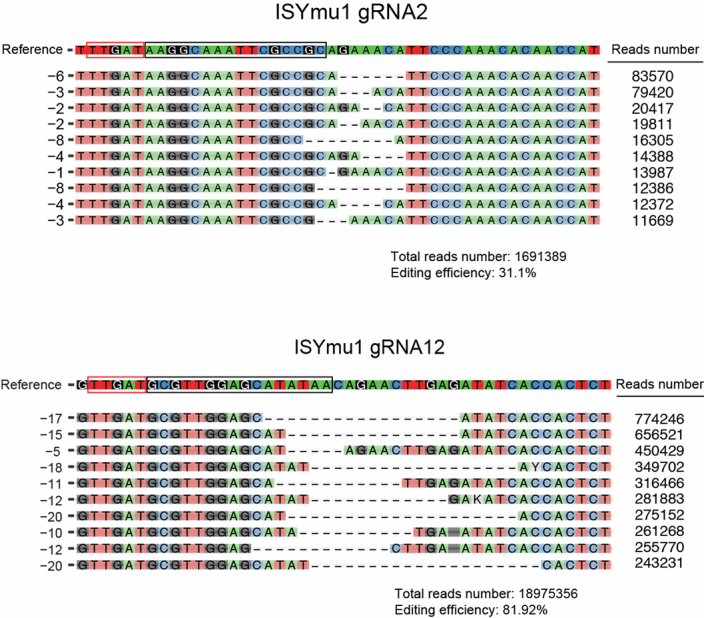
Representative DNA repair profiles for individual transgenic plants expressing either ISYmu1 *AtPDS3* gRNA2 or gRNA12. The top ten most common indel types are listed on the left. The read counts for each indel are listed on the right. The PAM is identified by the red box, and the target site is outlined by the black box, in the Reference sequence. The total read number and editing efficiency are listed below each indel profile. The name of each TnpB and gRNA is displayed above the Reference sequence for each indel repair profile.

To evaluate TnpB-mediated editing in transgenic plants we selected ISYmu1, as it demonstrated the highest average editing efficiency in *Arabidopsis* protoplast cells and was shown to exhibit no off-target editing in rice^19^. Two gRNAs with the most active editing were selected, each targeting a unique genomic context. gRNA2 targeted the coding region of *AtPDS3*, whereas gRNA12 targeted the promoter region directly upstream of the *AtPDS3* gene. Transgenic plants were created via standard floral dip transformation utilizing the same plasmids as for the protoplast experiments^23^. To test for sensitivity to temperature, transgenic plants expressing ISYmu1 were either grown at room temperature or subjected to a heat-shock treatment. We tested editing in wild-type (WT) plants, as well as in the *rna dependent rna polymerase 6* (*rdr6*) mutant which is known to have reduced transgene silencing^24^. Analysis using amp-seq revealed an average editing efficiency of 1.6% and 2.5% for gRNA2 in WT and *rdr6*, respectively (Fig. 1d). Analysis of gRNA12 revealed greater editing than gRNA2, averaging 44.9% editing in WT and 75.5% in *rdr6* (Fig. 1e). Comparison of editing efficiency in the plants grown at room temperature with those that received the heat-shock treatment revealed a preference for increased temperature for both target sites in the WT background, demonstrating 6.3-fold and 1.4-fold increases in editing for gRNA2 and gRNA12, respectively (Fig. 1d,e). In *rdr6*, we observed a 13-fold increase in editing for gRNA2, but little change in editing for gRNA12 (Fig. 1d,e). The editing outcomes from transgenic T1 plants expressing ISYmu1 consisted of chimaeric, deletion-dominant, DNA repair profiles (Extended Data Fig. 4). These data demonstrate that ISYmu1, encoded as a transgene, is capable of performing efficient genome editing in *Arabidopsis* plants, and that heat treatment and the *rdr6* silencing mutant can be used to increase editing efficiency.

**Fig. 2:**
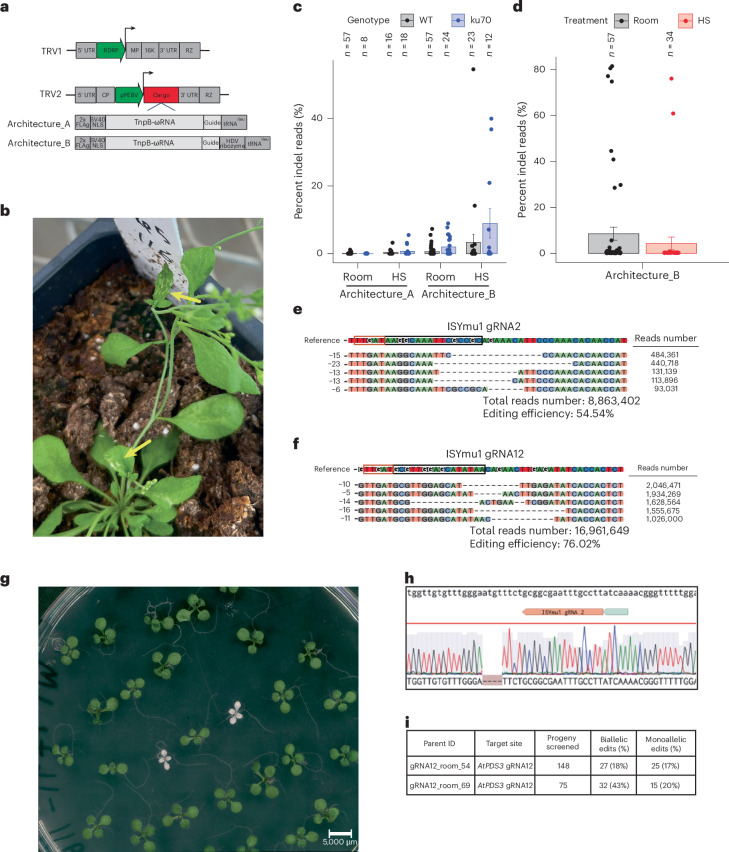
Somatic and heritable editing in *Arabidopsis* using TRV to deliver ISYmu1 TnpB and guide RNA targeting *AtPDS3.* **a**, Schematic of the TRV1 and TRV2 plasmids. Green arrows indicate the RNA-dependent RNA polymerase (RDRP) and pPEBV promoters for TRV1 and TRV2, respectively; the grey boxes in TRV1 and TRV2 indicate the native TRV components; the red Cargo box in TRV2 indicates the location of either Architecture_A or Architecture_B; below TRV2 are schematics of the components, Architecture_A or Architecture_B, cloned into the TRV2 Cargo slot. **b**, Representative picture of a plant displaying white sectors in leaves (yellow arrows) ~3 weeks after TRV delivery. **c**,**d**, Barplot displaying the somatic editing efficiencies (percent indel reads) (*Y* axis) for ISYmu1 gRNA2 in WT and *ku70* genetic backgrounds (**c**) and for ISYmu1 gRNA12 in WT (**d**). The TRV2 cargo architectures are plotted along the *X* axis with either room or HS treatment. Each dot represents an individual plant that underwent agroflood TRV delivery. The s.e.m. was calculated for each experiment. **e**,**f**, DNA indel repair profile for an individual WT plant that underwent delivery of TRV Cargo Architecture_B with ISYmu1 gRNA2 (**e**) or with ISYmu1 gRNA12 (**f**) under the heat-shock treatment. The top five most common indel types are listed on the left. The read counts for each indel are listed on the right. The PAM is identified by the red box, and the target site is outlined by the black box, in the Reference sequence. The total read number and editing efficiency are listed below each indel profile. **g**, Representative image of albino and green progeny seedlings from a WT plant showing 54.54% somatic editing using the TRV2 Architecture_B design with gRNA2 that underwent heat-shock treatment. **h**, Sanger sequencing trace file screenshot from one of the albino plants in Fig. 3a. Top: sequence of the wild-type reverse complement. Middle: the ISYmu1 gRNA2 target and PAM (grey box). Bottom: the ab1 trace file displaying a homozygous 4 bp deletion. **i**, Table summarizing the transmission of edited alleles from two individual plants that underwent agroflood delivery using ISYmu1 gRNA12. The ‘Progeny screened’ column indicates the number of seedlings genotyped; the ‘Biallelic edits (%)’ column indicates the number of seedlings containing biallelic edits; and the ‘Monoallelic edits (%)’ column indicates the number of plants harbouring monoallelic edits.

Encouraged by the ISYmu1 activity in transgenic *Arabidopsis* plants, we next tested ISYmu1 for TRV-mediated genome editing. TRV is a bipartite RNA virus composed of TRV1 and TRV2 (Fig. 2a). Previous work has shown that the TRV2 RNA can be engineered by inserting a cargo expression cassette downstream of the pea early browning virus promoter (pPEBV) (Fig. 2a)^25,26^. To test ISYmu1 for genome editing capabilities via TRV delivery to *Arabidopsis*, we engineered two TRV2 cargo architectures. In TRV2 Architecture_A, the tRNA^Ileu^ was directly downstream of the TnpB and gRNA sequences (Fig. 2a). In TRV Architecture_B, we included an HDV ribozyme sequence between the guide and tRNA^Ileu^ sequence (Fig. 2a). We included tRNA^Ileu^ in both designs as it was previously shown to promote systemic TRV movement and transmission of edited alleles to the next generation^25,26^.

First, we evaluated TRV-mediated editing potential with gRNA2 using both TRV2 Architecture_A and Architecture_B. gRNA2 was selected because it targets the *AtPDS3* coding sequence, enabling easy phenotypic screening for editing due to white photobleaching of cells containing biallelic mutations^25,26^. We delivered TRV vectors to both WT and the *ku70* genetic mutant. *Ku70* plays a role in the non-homologous end joining (NHEJ) double strand break repair pathway^27^. ISYmu1-mediated editing efficiency should be greater in the *ku70* genotype if double-stranded breaks generated by ISYmu1 are repaired through NHEJ. Each TRV2 plasmid was co-delivered with the TRV1 plasmid to *Arabidopsis* plants using the agroflood method^26^. White speckles were observed on some of the leaves at ~3 weeks post agroflooding, suggesting that sectors of cells contained biallelic mutations in the target *AtPDS3* gene (Fig. 2b). Amp-seq analysis revealed an average of 0.1% and 0% editing efficiency in leaf tissue of WT and *ku70* plants agroflooded with TRV2 Architecture_A and grown under room temperature, respectively (Fig. 2c). For the heat-shock-treated plants, we observed an average editing efficiency of 0.4% in WT and 0.7% in *ku70* plants agroflooded with TRV2 Architecture_A (Fig. 2c). Using TRV2 Architecture_B, we observed an average of 0.6% and 2% editing in WT and *ku70*, respectively, for the room-temperature-grown plants. For the plants that received TRV2 Architecture_B and a heat shock, we observed an average editing efficiency of 3.3% in WT and 8.9% in *ku70* (Fig. 2c). These results show that Architecture_B, containing the HDV ribozyme, generated higher editing than Architecture_A, and that the *ku70* mutant can enhance editing efficiency.

Next, we tested gRNA12 utilizing the TRV2 Architecture_B, since this architecture demonstrated the highest levels of editing for gRNA2. Using the same agroflood TRV delivery method to WT plants, we observed an average of 8.51% and 4.27% editing efficiency in room-temperature and heat-treatment growth conditions, respectively (Fig. 2d). Further, 6/57 plants displayed editing greater than 40%, with 4 plants showing greater than 75% editing when room-temperature treatment was used (Fig. 2d). Again, analysis of the repair outcomes showed deletion-dominant profiles for ISYmu1 gRNA2 and gRNA12 (Fig. 2e,f).

To test for transmission of edited alleles to the next generation, we first screened the progeny of a WT plant showing 54.54% somatic editing using the TRV2 Architecture_B design with gRNA2 that underwent heat-shock treatment. In total, 2,318 seeds were sown on ½ MS plates containing 3% sucrose. After 10 days, 68 albino seedlings were observed, suggesting biallelic mutations in the *PDS3* gene (Fig. 2g). To confirm that *AtPDS3* was mutated, we performed Sanger sequencing on the two white seedlings shown in Fig. 2g, which revealed both plants to be homozygous for a 4-bp frame-shift deletion (Fig. 2h). To further characterize transmission of edited alleles, amp-seq on 209 seedlings (41 albino and 168 green) showed that all of the albino seedlings contained biallelic mutations, with the majority of mutations being the 4-bp deletion observed in Fig. 2h (Supplementary Table 2). Of the 168 green seedlings, 8 were heterozygous (4-bp deletion in WT) (Supplementary Table 2).

**Extended Data Fig. 5:**
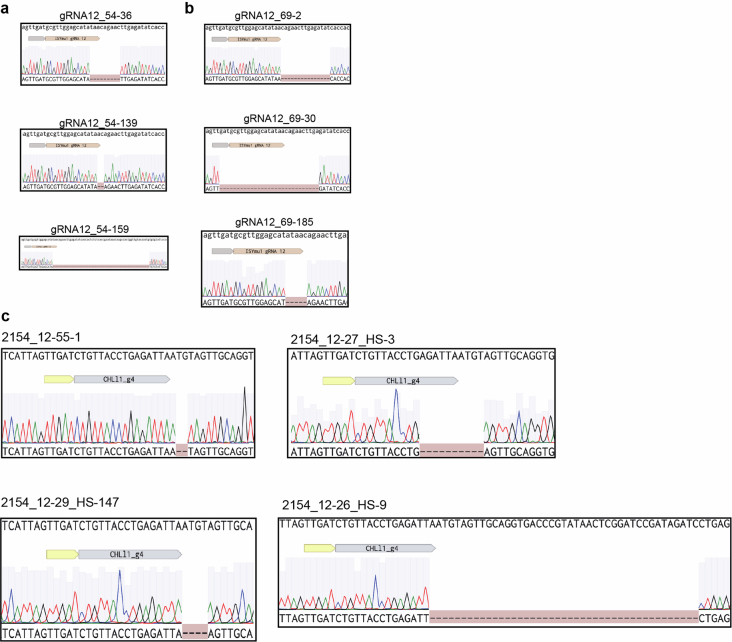
Sanger sequencing screenshots of the progeny plant genotypes. (**a, b**) Panels a and b correspond the *AtPDS3* gRNA12 targeted progeny from plants 54 and 69, respectively. The sequence at the top is the wild type genomic sequence; below that are the ISYmu1 gRNA12 target and PAM (gray box); the ab1 trace file displays the mutation. (**c**) Each box displays the genotype of a progeny plant from *AtCHLl1* gRNA4 editing experiment. The plant ID is indicated above each box. The sequence at the top is the wild type genomic sequence; below that are the ISYmu1 *AtCHLl1* gRNA4 target and PAM (yellow box); the ab1 trace file displays the mutation.

Next, we characterized transmission of edited alleles from two individual lines, plant 54 (80.5% somatic editing) and plant 69 (77.1% somatic editing), that underwent agroflood using gRNA12 TRV2 Architecture_B with the room-temperature condition. As expected, we did not observe any albino seedlings, probably because this target site is located upstream of the *AtPDS3* transcription start site. Using Sanger sequencing, we analysed the genotypes of 148 and 75 progeny seedlings from plants 54 and 69, respectively. Sanger sequencing analysis of the progeny from plant 54 revealed 27 (18%) biallelic and 25 (17%) monoallelic edited plants (Fig. 2i, Supplementary Table 2 and Extended Data Fig. 5a)^28^. For plant 69, we observed higher transmission of edited alleles, totalling 32 (43%) biallelic and 15 (20%) monoallelic edited plants (Fig. 2i, Supplementary Table 2 and Extended Data Fig. 5b)^28^. These data demonstrate the heritability of edits generated via TRV delivery of ISYmu1 at two distinct target sites.

**Fig. 3:**
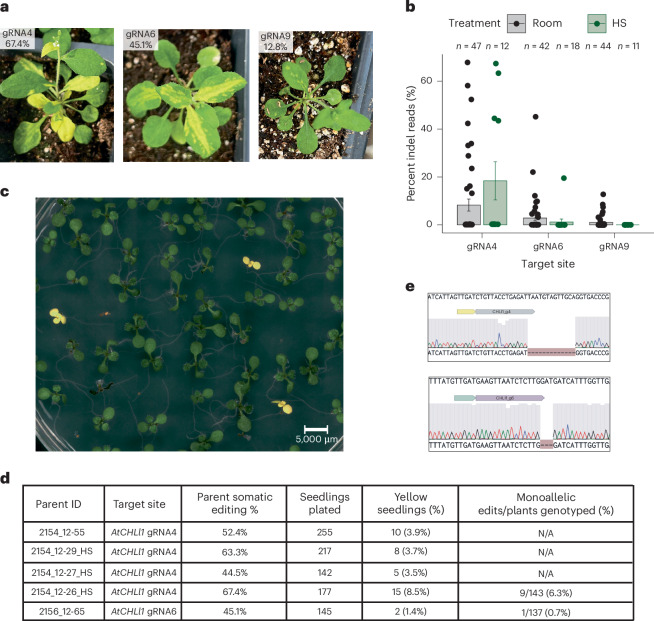
Somatic and heritable editing in *Arabidopsis* using TRV to deliver ISYmu1 TnpB and guide RNA targeting *AtCHLl1.* **a**, Representative pictures of plants displaying yellow sectors ~2 weeks after TRV delivery. The gRNA and somatic editing efficiency is indicated in the upper left corner of each picture. **b**, Barplot displaying the somatic editing efficiencies (±s.e.m.) for ISYmu1 gRNA4, gRNA6 and gRNA9 in WT. The gRNA target site is plotted along the *X* axis. The *Y* axis indicates the editing efficiencies (percent indel reads). Each dot represents an individual plant that underwent agroflood TRV delivery. **c**, Representative image of yellow and green progeny seedlings from a WT plant showing 67.4% somatic editing using the TRV2 Architecture_B design with gRNA4 that underwent heat-shock treatment. **d**, Table summarizing the transmission of edited alleles from four and one individual plants that underwent agroflood delivery using ISYmu1 gRNA4 and gRNA6, respectively. **e**, Representative Sanger sequencing trace file screenshots from a yellow plant harbouring an edit at gRNA4 (top) or gRNA6 (bottom). For each panel: top, wild-type sequence; middle, the ISYmu1 gRNA target and PAM; bottom, the ab1 trace file displaying a homozygous deletion.

To test the applicability of this approach to another locus, we designed six ISYmu1 gRNAs targeting the *AtCHLl1* gene (AT4G18480) (Supplementary Table 1). *AtCHLl1* was chosen due to the obvious yellow phenotypic readout that *AtCHLl1* homozygous mutant plants display^29^. The agroflood method was used to individually deliver TRV Architecture_B vectors targeting each of the six *AtCHLl1* sites. Plants were exposed to either room temperature or heat-shock growth conditions as previously described. About 2 weeks post agroflood, we observed yellow sectors on some of the plants infected with TRV ISYmu1 gRNA4, gRNA6 and gRNA9 (Fig. 3a). To quantify somatic editing, tissue samples from plants infected with TRV targeting gRNA4, gRNA6 and gRNA9 were collected for amp-seq analysis. We observed an average of 8.3%, 2.9% and 1% somatic editing for gRNA4, gRNA6 and gRNA9 for the room-temperature condition, respectively (Fig. 3b). For the plants that underwent the heat shock, we detected an average editing frequency of 18.4%, 1.2% and 0% for gRNA4, gRNA6 and gRNA9, respectively (Fig. 3b). Further, 4/47 (8.5%) and 4/12 (33.3%) plants infected with ISYmu1 gRNA4 displayed somatic editing greater than 40% for room-temperature and heat-shock samples, respectively (Fig. 3b).

Next, we screened the progeny from plants infected with TRV targeting gRNA4 and gRNA6 to quantify transmission of edited alleles. Seedlings were grown on ½ MS plates containing 3% sucrose, and after 10 days we observed yellow seedlings, consistent with the biallelic mutation genotype of this gene (Fig. 3c)^29^. In total, we observed 3.5–8.5% yellow progeny from plants infected with TRV ISYmu1 gRNA4 (Fig. 3d). For the progeny of a plant infected with gRNA6, we observed fewer yellow seedlings, totalling 2/145 (1.4%) (Fig. 3d). Sanger sequencing revealed that all of the yellow plants harboured biallelic mutations at the gRNA4 or gRNA6 target site (Fig. 3e, Extended Data Fig. 5c and Supplementary Table 2). Next, Sanger sequencing revealed that 9/143 (6.3%) and 1/137 (0.7%) green seedlings from plants 2154_12-26_HS (gRNA4) and 2156_12-65 (gRNA6), respectively, contained monoallelic edits (Fig. 3d and Supplementary Table 2). These data indicate that TRV-mediated editing with ISYmu1 is capable of generating targeted somatic mutations at three target sites of the *AtCHLl1* gene, and that edited alleles can be transmitted to the next generation.

**Extended Data Fig. 6:**
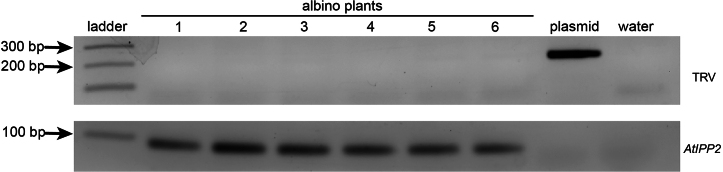
RT-PCR gel showing absence of the TRV in the progeny of a TRV-infected plant. RT-PCR was performed using total RNA extracted from albino plants homozygous for a 4 bp deletion at the *AtPDS3* gene. Gel electrophoresis image of RT-PCR performed using primers targeting TRV (upper panel) and *AtIPP2* (lower panel). The lanes are indicated (from left to right) as ladder, six individual albino plants, plasmid control, and a water control. Black arrows indicate the amplicon size in base pairs (bp). Source data

It has been demonstrated that TRV is not transmitted to the next generation following agroflood inoculation of plants^25,26^. To confirm that the TRV was not present in the progeny of a TRV-infected plant, RT–PCR was performed on 5 albino plants harbouring homozygous 4-bp deletions at *AtPDS3*. Consistent with the literature, TRV was not detected in any of the albino plants (Extended Data Fig. 6)^25,26^. These data indicate that TRV-mediated biallelic edits using ISYmu1 are heritable and virus-free.

**Extended Data Fig. 7:**
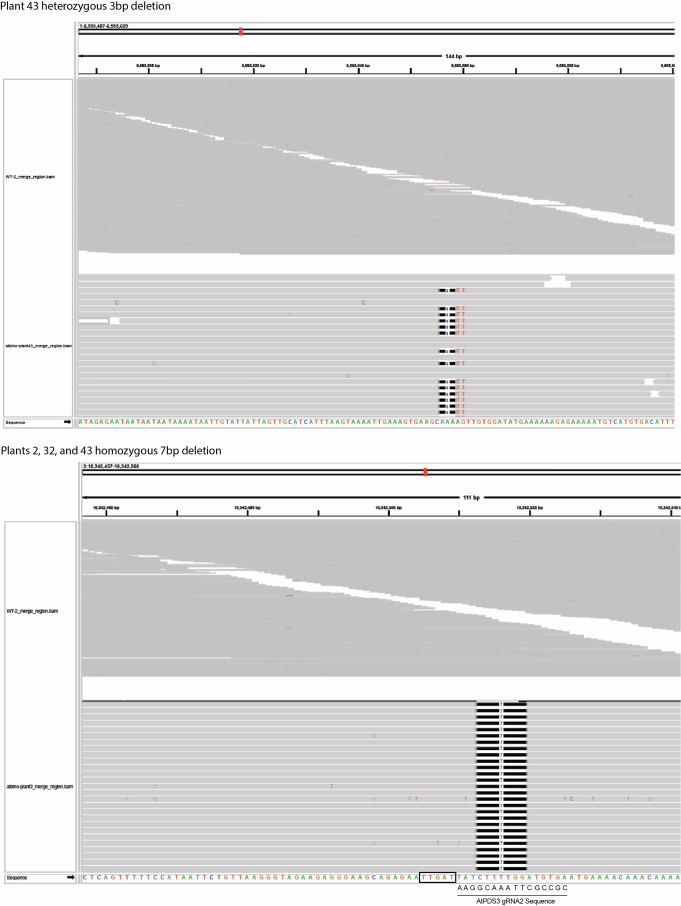
Potential off-target mutations identified by whole genome sequencing. Genome browser screenshot of the potential edits identified by whole genome sequencing for Plant 43 (heterozygous 3 bp deletion) and Plants 2, 32, and 43 (homozygous 7 bp deletion). The mutation is displayed on the bottom track, and the wild type sequence is displayed on the top track of each screenshot. The plant ID, zygosity, and mutation type is displayed above each screenshot. The genomic location is listed in the upper left corner of each screenshot.

**Extended Data Fig. 8:**
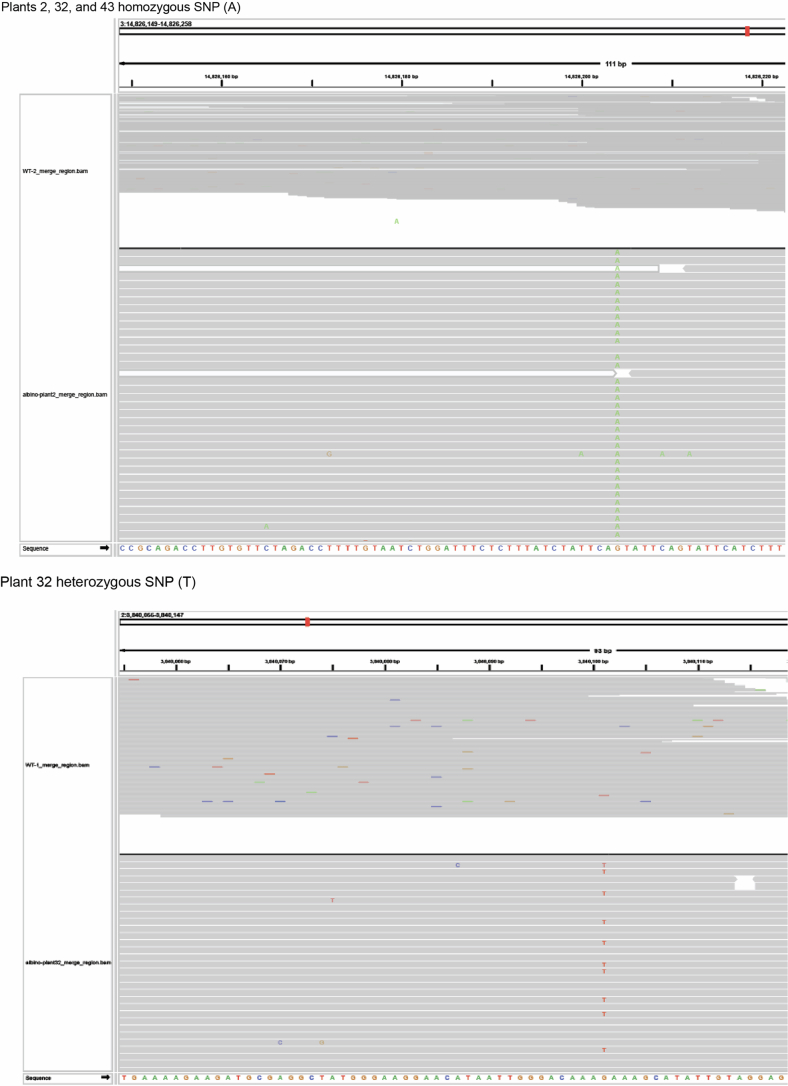
Potential off-target mutations identified by whole genome sequencing. Genome browser screenshot of the potential edits identified by whole genome sequencing for Plants 2, 32, and 43 (homozygous SNP (A)) and Plant 32 (heterozygous SNP (T)). The mutation is displayed on the bottom track, and the wild type sequence is displayed on the top track of each screenshot. The plant ID, zygosity, and mutation type is displayed above each screenshot. The genomic location is listed in the upper left corner of each screenshot.

**Extended Data Fig. 9:**
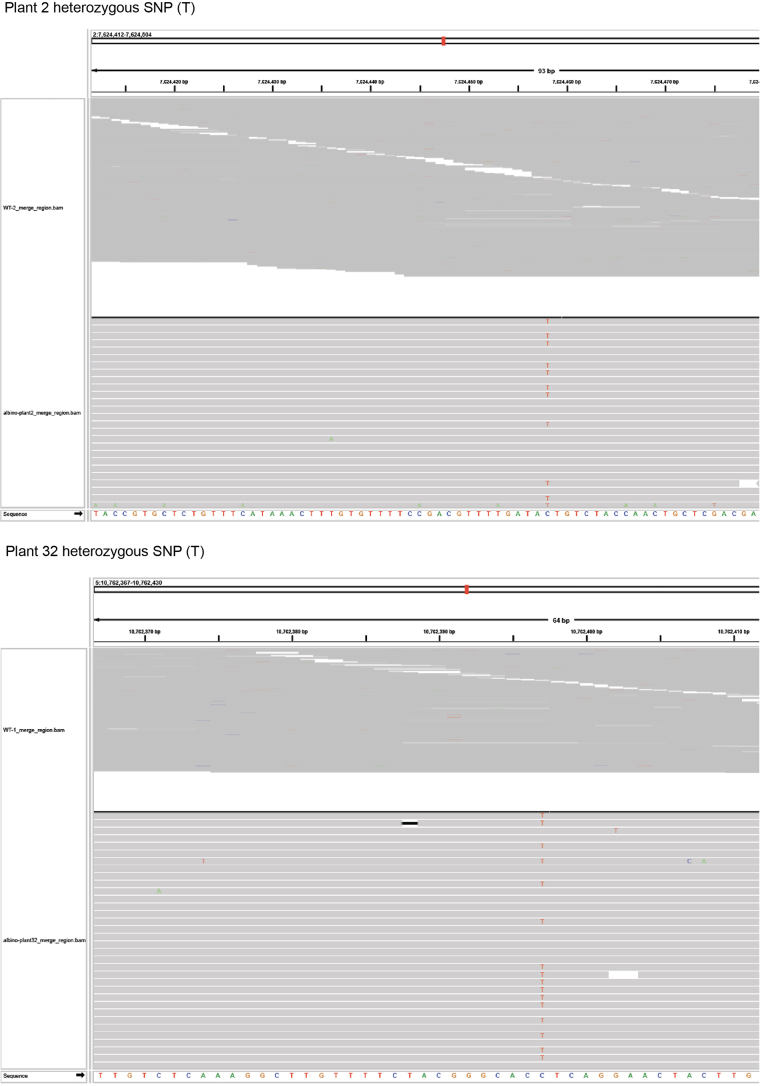
Potential off-target mutations identified by whole genome sequencing. Genome browser screenshot of the potential edits identified by whole genome sequencing for Plant 2 (heterozygous SNP (T)) and Plant 32 (heterozygous SNP (T)). The mutation is displayed on the bottom track, and the wild type sequence is displayed on the top track of each screenshot. The plant ID, zygosity, and mutation type is displayed above each screenshot. The genomic location is listed in the upper left corner of each screenshot.

**Extended Data Fig. 10:**
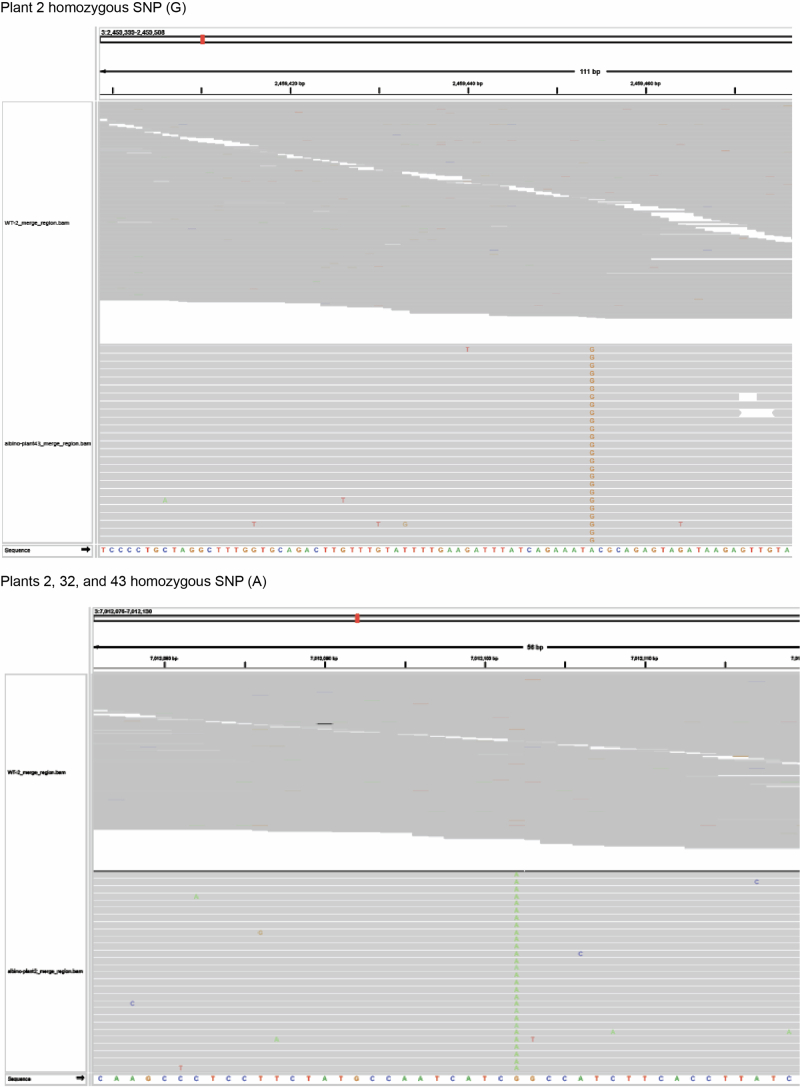
Potential off-target mutations identified by whole genome sequencing. Genome browser screenshot of the potential edits identified by whole genome sequencing for Plant 2 (homozygous SNP (G)) and Plants 2, 32, and 43 (homozygous SNP (A)). The mutation is displayed on the bottom track, and the wild type sequence is displayed on the top track of each screenshot. The plant ID, zygosity, and mutation type is displayed above each screenshot. The genomic location is listed in the upper left corner of each screenshot.

To evaluate off-target editing, we surveyed 3 individual albino plants harbouring biallelic mutations generated by ISYmu1 TRV2 Architecture_B gRNA2. Whole-genome sequencing was performed to generate an average of 770× coverage, with greater than 99% of the genome covered by mapped reads (Supplementary Table 3). In all 3 samples, we confirmed the targeted mutations in the *AtPDS3* gene, as previously identified using amp-seq. In addition, we found a large number of variant differences compared with the Col-0 reference genome both in the control and the edited plants (Supplementary Table 4), suggesting that most of the variants detected are due to spontaneous mutations present in our lab strain of *Arabidopsis*. To screen for variants potentially caused by ISYmu1 off-target editing, all variants in the edited plants were filtered with variants already present in the control background. Variants with coverage lower than 30-fold were also filtered out. The remaining variants were checked manually for any false positive variant calling. In the 3 albino plants we sequenced, only 5, 5 and 4 variants were detected, and these variants are all outside the predicted potential off-target sites based on sequence similarity to the *AtPDS3* gRNA2 sequence (Extended Data Figs. 7–10, and Supplementary Tables 4 and 5)^30^. In line with ISYmu1 off-target analysis reported in rice and human cells^19,20^, these data further demonstrate the high target-site specificity of ISYmu1.

A long-term goal of plant scientists has been the development of fast and easy means of editing plant genomes without the need for tissue culture and transgenesis. Very recently, low levels of tissue-culture-free heritable gene editing was demonstrated by delivering Cas9 and the gRNA to *Nicotiana benthamiana* using the tobacco ringspot virus (TRSV)^31^. They improved heritability by co-delivering the Cas9-gRNA TRSV with an *rdr6* virus-induced gene silencing knockdown sequence on the apple latent spherical virus (ALSV)^31^. Here we developed a streamlined and easy-to-use approach utilizing the ultracompact site-specific TnpB genome editor, ISYmu1, together with tobacco rattle virus, for heritable plant genome editing. These results should accelerate high-throughput genome editing for both basic and applied research. We anticipate this approach to be applicable to other novel TnpBs, various viral vectors and a number of plant species for genome editing. Recent work has uncovered many TnpB systems from diverse microbial sources, including enzymes with unique protospacer adjacent motif (PAM) sequence specificities^32^, which can increase the range of target DNA sequences that could be edited using this approach. The TRV virus used in this study has a broad host range of over 400 species, including many solanaceous plants such as tomato, ornamental plants and other crops^33^. In addition, plant viruses with similar cargo capacities, such as potato virus X and barley stripe mosaic virus, are likely to be amenable to this approach since it has been demonstrated that they are capable of viral-mediated heritable gene editing by delivering the gRNA to a Cas9-expressing transgenic plant^34^. Further, because this approach can create sectors of tissue harbouring somatic biallelic edits, it may also serve as a tool to enable the study of genes that cause embryonic lethality or severe pleiotropic effects as homozygous mutants. Finally, in addition to being an important tool for crop biotechnology, viral delivery of TnpBs could enable high-throughput CRISPR screens in model plant species such as *Arabidopsis*, further unlocking their potential for genetic discovery.

## Methods

### Plasmids used in this study

Plasmids used for bacterial assay were generated as follows. The single expression cassette containing TnpB and ωRNA sequences were synthesized as geneblocks from Integrated DNA Technologies (IDT) and were golden-gate cloned using BsmbI restriction enzyme (E1602S) into a vector (chloramphenicol resistance) under a single tetracycline-inducible promoter (TetR/pTet) to make the TnpB-ωRNA plasmid. Target sites with various PAM sequences and target sites were golden-gate cloned with BbsI restriction enzyme (R3539S) into a vector (ampicillin/carbenicillin resistance).

Plasmids were generated for protoplast and floral dip experiments in a two-step cloning strategy. In step one, the ISDra2, ISYmu1 and ISAam1 protein coding sequences and their ωRNAs were synthesized as geneblocks by IDT. Then, starting with the pC1300_pUB10_pcoCASphi_E9t_MCS_version2 vector^35^, we used NEBuilder HiFi DNA Assembly (catE2621) and PCR to assemble the TnpB-ωRNA geneblocks into plant expression vectors with a toxic *ccdB* insert flanked by PaqCI sites immediately downstream of the ωRNA scaffold and preceding an HDV ribozyme sequence. The HiFi reactions were then transformed into One Shot *ccdB* Survival 2 T1R Competent Cells (A10460) to obtain the pMK003 (ISDra2), pMK025 (ISYmu1) and pMK024 (ISAam1) intermediate vectors for facile guide sequence cloning (Supplementary Table 6). In step two, guide sequences were synthesized as individual top and bottom strands with 4 base pair overhangs from IDT, phosphorylated and annealed, and then used for golden-gate assembly using the NEB PaqCI (R0745) enzyme (Supplementary Table 7). When transformed into NEB 10-beta competent *E. coli* (C3019), vectors that still contained the *ccdB* gene would kill the cells, leaving behind only the transformants possessing successfully assembled TnpB plant expression vectors harbouring a guide RNA sequence.

TRV vectors targeting *AtPDS3* were created with the pDK3888 TRV2 plasmid as a base vector^26^. NEBuilder HiFi DNA Assembly (E2621) was used to clone the ISYmu1 gRNA2 Architecture_A, ISYmu1 gRNA2 Architecture_B and ISYmu1 gRNA12 Architecture_B into the TRV2 cargo slot. First, pDK3888 was digested using NEB ZraI (R0659), NEB PmlI (R0532) and NEB Quick CIP (M0525) overnight, and purified using Qiagen QiaQuick purification column (28104). Next, three PCR reactions were performed to amplify the fragments needed for NEBuilder HiFi DNA Assembly, followed by purification using a Qiagen QiaQuick purification column (28104) (Supplementary Table 8). Then, the digested and purified pDK3888 plasmid and purified PCR fragments were used to assemble the final TRV2 plasmid using NEBuilder HiFi DNA Assembly according to manufacturer protocol. Finally, the NEBuilder HiFi DNA Assembly reaction was transformed into NEB 10-beta competent *E. coli* (C3019). TRV2 vectors targeting *AtCHLl1* were created using golden-gate assembly^36^. Two oligos corresponding to the target site were phosphorylated and annealed. Then, the annealed double-stranded DNA was used in a PaqCI (R0745S) golden-gate reaction with the pMK435 *ccdb* intermediate vector (Supplementary Table 8). The golden-gate reaction was then transformed into NEB 10-beta competent *E. coli* (C3019). Correct plasmids were confirmed using Primordium whole-plasmid sequencing. Plasmids and their descriptions can be found in Supplementary Table 6.

### Bacterial interference assay

For the bacterial interference assay, we co-transfected 100 ng of the TnpB-ωRNA plasmid and 100 ng of the target plasmid to 33 µl of NEB 10-beta electrocompetent *E. coli* cells (C3020K). Specifically, the target plasmid contains a target site either flanking the canonical PAM (TTGAT for ISYmu1 and ISDra2 and TTTAA for ISAam1) or flanking a non-canonical PAM (GGGGG). The cells were recovered in 1 ml of NEB 10-Beta Stable/Outgrowth media (B9035S) for 1 h. Following recovery, a series of 5-fold dilutions of the recovery culture were prepared. Each dilution (5 μl) was spot plated onto LB-agar plates containing double antibiotics (34 μg ml^−1^ chloramphenicol, 100 μg ml^−1^ carbenicillin and 2 nM anhydrotetracycline) and onto control plates with a single antibiotic (34 μg ml^−1^ chloramphenicol and 2 nM anhydrotetracycline). If no colonies were visible on the serial dilution plates, 400 μl of the 1 ml recovery culture was plated entirely on the double antibiotic plate to enhance detection sensitivity. Plates were left overnight at either 26 °C or 37 °C, and colony-forming units (c.f.u.s) were counted on all plates the next morning. The normalized c.f.u.s were calculated by taking the ratio of c.f.u.s on the double antibiotic plates to the c.f.u.s on the single antibiotic plates. The normalized c.f.u.s in the canonical PAM conditions were compared to those in the non-canonical PAM conditions. Experiments were performed in triplicate.

### Plant materials and growth conditions

For protoplast preparation, *Arabidopsis* Columbia ecotype (Col-0) seeds were suspended in a 0.1% agarose solution and kept at 4 °C in the dark for 3 days to stratify. Following stratification, seeds were planted on Jiffy pucks and grown under a 12-h/12-h light/dark photoperiod with low-light condition at 20 °C for 3–4 weeks^22^.

For the creation of transgenic plants, the *Arabidopsis* Col-0 ecotype was used. The *ku70* (SALK_123114) genotype was obtained from Feng Zhang lab at the University of Minnesota. The *rdr6* genotype was created using CRISPR-Cas9, resulting in a 616-bp deletion in the gene body of *rdr6*. Floral dip transformation was performed according to the protocol as previously outlined using the Agl0 *Agrobacterium* strain^23^. Transgenic T1 plants were screened using ½ MS plates with 40 μg ml^−1^ hygromycin B under a 16-h/8-h light/dark cycle at 23 °C. After 1 week, transgenic seedlings that passed selection were transferred to soil and moved to a greenhouse (23 °C) for the rest of their life cycle.

For agroflood experiments, sterilized seeds were sown on ½ MS agar plates and stratified for 5 days. After 5 days, the seeds were moved to a growth room and grown under a 16-h/8-h light/dark cycle at 23 °C for 8–10 days. The seedlings were then used for TRV delivery.

A subset of transgenic T1 plants and plants that underwent agroflood were subjected to a heat-shock treatment modified from ref. ^37^. Seedlings that passed selection or underwent agroflood were then transplanted to soil and grown in a greenhouse (23 °C) for 1 week. After 1 week, plants that did not receive a heat-shock treatment continued to grow in the greenhouse (23 °C); however, plants that underwent heat-shock treatment were exposed to 8 h (9:00–17:00) of heat exposure at 37 °C every day for 5 days, followed by 2 days of recovery at a greenhouse (23 °C). This heat-shock regime lasted for 2 weeks.

### Protoplast isolation and transfection

*Arabidopsis* mesophyll protoplast isolation was performed as previously described^22^. Plasmid transfections into *Arabidopsis* protoplasts were performed using 20 µg of plasmid following ref. ^35^. The concentrations of plasmids were determined using a nanodrop spectrophotometer. Plasmids were added to the bottom of each transfection tube, and the volume of the plasmids was supplemented with water to reach 20 µl. Protoplasts (200 µl) were added, followed by 220 µl of fresh and sterile polyethylene glycol (PEG)-CaCl_2_ solution. The samples were mixed by gently tapping the tubes and incubated at room temperature for 10 min. After 10 min, 880 µl of W5 solution was added and mixed with the protoplasts by inverting the tube two to three times to stop the transfection. Next, protoplasts were collected by centrifuging the tubes at 100 relative centrifugal force (RCF) for 2 min and resuspended in 1 ml of WI solution. The protoplast cells were then plated in 6-well plates precoated with 5% calf serum. Protoplast cells in the 6-well plates were incubated at 26 °C for 48 h. During the 48-h incubation, the protoplast cells were subjected to a 37 °C heat-shock treatment for 2 h at 16 h post transfection. At 48 h post transfection, protoplasts were collected for genomic DNA extraction.

### TRV delivery to *Arabidopsis* seedlings

TRV delivery was performed as previously described^26^. TRV1 and TRV2 vectors were first introduced into the GV3101 *Agrobacterium* strain. The *Agrobacterium* harbouring TRV vectors were then grown in 200 ml of lysogeny broth (LB) with antibiotics for 18 h at 28 °C. *Agrobacterium* cultures were centrifuged for 20 min at 3,500 × *g*. The LB was discarded and the *Agrobacterium* cells were resuspended in 200 ml of sterile water. The resuspended *Agrobacterium* was centrifuged for 10 min at 2,109 × *g*. The supernatant was discarded and the pellet was resuspended in sterile agro-infiltration buffer containing 10 mM MgCl_2_, 10 mM 2-(*N*-morpholino) ethanesulfonic acid and 250 µM acetosyringone to otical density (OD)_600_ = 1.5. The *Agrobacterium* cells were then incubated at 23 °C for 3 h with slow shaking. After 3 h, the *Agrobacterium* harbouring TRV1 and TRV2 were mixed in a 1:1 ratio, and 15 ml of this 1:1 mixture of TRV was delivered to seedlings at 8–10 days old. After 4 days of agroflood co-culture, seedlings were transplanted to soil.

### Screening the progeny of TRV-infected plants for edits

Seeds were harvested from the TRV-infected plants ~12 weeks after TRV delivery. The seeds were sown on ½ MS plates supplemented with 3% sucrose and stored at 4 °C in the dark for 5 days to stratify. After 5 days, the seeds were moved to a growth room and grown under a 16-h/8-h light/dark cycle at 23 °C for 10–12 days. Next, a subset of plants was sampled for genotyping. A single piece of leaf tissue was sampled, and DNA was extracted using Invitrogen Platinum Direct PCR Universal Master Mix (A44647500) according to manufacturer instructions. The DNA was then used for amp-seq or Sanger sequencing using primers listed in Supplementary Table 9.

### Next-generation amplicon sequencing

DNA was extracted from protoplast samples with Qiagen DNeasy plant mini kit (Qiagen, 69106). Tissue was collected from transgenic plants by sampling and pooling leaf tissue from 3 random leaves on a single plant 3 weeks after being transplanted to soil. For the plants that underwent agroflood, leaf tissue was sampled by collecting and pooling tissue from 3 random (however, if white or yellow sectors were visible, they were sampled) leaves on a single plant distal to the TRV delivery site 3 weeks after being transplanted to soil. Once tissue samples were collected, they were frozen at −80 °C overnight. The samples were then ground and DNA was extracted using the Invitrogen Platinum Direct PCR Universal Master Mix (A44647500) according to manufacturer instructions. For the progenies of plants that underwent agroflood, a single leaf tissue was sampled and DNA was extracted using Invitrogen Platinum Direct PCR Universal Master Mix (A44647500) according to manufacturer instructions. The DNA was then used for next-generation amplicon sequencing.

Following ref. ^35^, editing efficiency was characterized using single-end next-generation sequencing on the Illumina NovaSeqX platform. Libraries were prepared via a 2-step PCR amplification method. In the first round of amplification, each target site was amplified using primers flanking the target site (Supplementary Table 9). After 25 cycles of amplification, the reactions were cleaned using 1.0× Ampure XP bead purification (Beckman Coulter, A63881). Next, each sample went through 12 additional cycles of amplification using Illumina indexing primers. The samples were cleaned using 0.7× Ampure XP bead purification. Samples were checked for purity on a 2% agarose gel, quantified using a nanodrop spectrophotometer, normalized and pooled.

### Next-generation amplicon sequencing analysis

Amplicon sequencing analysis was performed following ref. ^35^. Single-end reads were used for analysis. Reads were adapter trimmed using Trim Galore default settings. Remaining reads were mapped to the target genome region using the BWA aligner (v.0.7.17, BWA-MEM algorithm). Sorted and indexed bam files were used as input files for further analysis using the CrispRvariants R package (v.1.14.0). Each mutation pattern with corresponding read counts was exported using the CrispRvariants R package. After assessing all control samples, a criterion to classify reads as edited was established: only reads with a ≥3-bp deletion or insertion (indel) of the same pattern (indels of same size starting at the same location) with ≥10 read counts from a sample were counted as edited reads. Single nucleotide variants were also filtered out.

### Off-target analysis

Off-target analysis was performed as previously described^35^. DNA from single *Arabidopsis* seedlings was extracted with the Qiagen DNeasy plant mini kit and sheared to 300-bp size with a Covaris sonicator. Library preparation was performed with a Tecan Ovation Ultralow V2 DNA-seq kit. For variant calling, WGS reads were aligned to the TAIR10 reference genome using BWA mem (v.0.7.17)^38^ with default parameters. GATK (4.2.0.0)^39^ MarkDuplicatesSpark was used to remove PCR duplicate reads. Then GATK HaplotypeCaller was used to call raw variants. Raw single nucleotide polymorphisms (SNPs) were filtered with QD < 2.0, FS > 60.0, MQ < 40.0 and SOR > 4.0. Raw InDels were filtered with QD < 2.0, FS > 200.0 and SOR > 10.0 and used for base quality score recalibration. The recalibrated bam was further applied to GATK and Strelka (v.2.9.2) SNPs/InDel calling. Only SNPs/InDels called by both GATK and Strelka were used for further filtering. The intersection of SNPs/InDel called by GATK with Strelka (v.2.9.2)^40^ was obtained using BedTools (v.2.26.0)^41^. SNPs/InDel were filtered with wild-type background using BedTools (v.2.26.0). Variants with depth coverage lower than 30 were filtered.

### RT–PCR

Total RNA from TRV-infected progeny plants was extracted using Zymo Research Direct-zol RNA MiniPrep kit (R2052). Total RNA was converted to cDNA using the Invitrogen SuperScript IV VILO Master Mix (11766050). The RT–PCR control was performed using primers targeting the *AtIPP2* gene (Supplementary Table 10). PCR was performed to check for the presence/absence of the TRV vector using SP9238 and SP9239 (Supplementary Table 10)^26^. PCR was performed with New England Biolabs Q5 High-Fidelity 2× Master Mix (M0492L) according to manufacturer instructions, using 2 µl of cDNA in a 25-µl reaction. PCR conditions included a 98 °C initial denaturation step for 30 s, 35×(98 °C, 10 s; 55 °C, 20 s; 72 °C, 10 s) and 72 °C for 2 min. PCR amplicons (10 µl) were analysed using 2% agarose gel electrophoresis.

### Reporting summary

Further information on research design is available in the Nature Portfolio Reporting Summary linked to this article.

## Supplementary information

Supplementary Information Supplementary Tables 1–10.

Reporting Summary

## Source data

Source Data Extended Data Fig. 6 Unprocessed RT–PCR gel.

## Data Availability

All the amp-seq data generated in this study are accessible at NCBI Sequence Read Archive under BioProject PRJNA1124592. Whole-genome sequencing data are accessible at BioProject PRJNA1146711. Source data are provided with this paper.
