# Supplementary material for: Viral delivery of an RNA-guided genome editor for transgene-free germline editing in Arabidopsis
Source: Nat Plants. 2025 Apr 22;11(5):967–76. doi: 10.1038/s41477-025-01989-9 (PMC12095077; doi:10.1038/s41477-025-01989-9)
Supplement: Supplementary file 1 — Supplementary Tables 1–10. [file 41477_2025_1989_MOESM1_ESM.pdf]

nature plants

Supplementary information https://doi.org/10.1038/s41477-025-01989-9

Viral delivery of an RNA-guided genome editor for transgene-free germline editing in *Arabidopsis*

In the format provided by the authors and unedited

Supplementary Tables

TnpB	Guide	gRNA sequence	Genomic location	Gene
ISDra2	gRNA1	ttacgaattgatgaccatat	4:8195186-8195206	*PDS3*
ISDra2	gRNA2	aaggcaaattcgccgcagaa	4:8194730-8194749	*PDS3*
ISDra2	gRNA3	tcacattaagcctagaaact	4:8193921-8193940	*PDS3*
ISDra2	gRNA4	cccaagttctccaaataaat	4:8193476-8193495	*PDS3*
ISDra2	gRNA5	tacccatcctaaagtatggg	4:8192944-8192963	*PDS3*
ISDra2	gRNA7	aaattcaacatctttctcta	4:8190835-8190854	*PDS3*
ISDra2	gRNA8	tggtctcactttccgaatta	4:8194167-8194186	*PDS3*
ISDra2	gRNA9	agctttgaaccggtttcttc	4:8192305-8192324	*PDS3*
ISDra2	gRNA11	cgaaaactgaagaacacata	4:8191494-8191513	*PDS3*
ISDra2	gRNA12	gcgttggagcatataacaga	4:8195556-8195575	*PDS3*
ISDra2	gRNA13	taaagagaggaaattgcagg	4:8194513-8194532	*PDS3*
ISDra2	gRNA14	ggtagagctgataagatata	4:8193625-8193644	*PDS3*
ISDra2	gRNA15	aaaattggattaatgtgcac	4:8193203-8193222	*PDS3*
ISDra2	gRNA16	tactattaaatgtcaaaatc	4:8193077-8193096	*PDS3*
ISDra2	gRNA17	caatacaaataaatacatgc	4:8193117-8193136	*PDS3*
ISDra2	gRNA18	caattcaagctaattataga	4:8193095-8193114	*PDS3*
ISDra2	gRNA19	gagcttaacttggtagagta	4:8192577-8192596	*PDS3*
ISDra2	gRNA20	ttgtcagctttcttatggat	4:8192528-8192547	*PDS3*
ISDra2	gRNA21	gttgattaacttgtactacc	4:8192131-8192150	*PDS3*
ISDra2	gRNA22	taacttgtactacctcatcc	4:8192125-8192144	*PDS3*
ISYmu1	gRNA1	ttacgaattgatgacc	4:8195187-8195202	*PDS3*

ISYmu1	gRNA2	aaggcaaattcgccgc	4:8194730-8194745	*PDS3*
ISYmu1	gRNA3	tcacattaagcctaga	4:8193921-8193936	*PDS3*
ISYmu1	gRNA4	cccaagttctccaaat	4:8193476-8193491	*PDS3*
ISYmu1	gRNA5	tacccatcctaaagta	4:8192944-8192959	*PDS3*
ISYmu1	gRNA7	aaattcaacatctttc	4:8190839-8190854	*PDS3*
ISYmu1	gRNA8	tggtctcactttccga	4:8194171-8194186	*PDS3*
ISYmu1	gRNA9	agctttgaaccggttt	4:8192309-8192324	*PDS3*
ISYmu1	gRNA10	taacttgtactacctc	4:8192129-8192144	*PDS3*
ISYmu1	gRNA12	gcgttggagcatataa	4:8195560-8195575	*PDS3*
ISAam1	gRNA11	caattcatctggtatc	4:8191755-8191770	*PDS3*
ISAam1	gRNA12	ccaagaacaagcctta	4:8191347-8191362	*PDS3*
ISAam1	gRNA13	gttttgtcctcttctc	4:8190412-8190427	*PDS3*
ISAam1	gRNA14	caatttacctatctta	4:8193797-8193812	*PDS3*
ISAam1	gRNA15	cacataattgaaaaga	4:8194919-8194934	*PDS3*
ISAam1	gRNA16	aatttgttacacaact	4:8195755-8195770	*PDS3*
ISAam1	gRNA17	tttgtgtggtatttaa	4:8195158-8195173	*PDS3*
ISYmu1	gRNA3	CCAAAGATTGGTGGTG	4:10202829-10202844	*CHLI1*
ISYmu1	gRNA4	CTGTTACCTGAGATTA	4:10202754-10202769	*CHLI1*
ISYmu1	gRNA5	CTTCCTTTGGGTGCAA	4:10202610-10202625	*CHLI1*
ISYmu1	gRNA6	GAAGTTAATCTCTTGG	4:10202481-10202496	*CHLI1*
ISYmu1	gRNA7	GTTCTTTTGGATTCAG	4:10202448-10202463	*CHLI1*
ISYmu1	gRNA9	CGGTTTGGTATGCATG	4:10202313-10202328	*CHLI1*

Supplementary Table 1: ISDra2, ISYmu1 and ISAam1 target sites. The table consists of four columns. The TnpB and Guide columns indicate the TnpB gRNA used. The gRNA sequence column lists the gRNA sequence used for targeted genome editing. The Genomic location column provides the location of each gRNA target site in the *Arabidopsis* genome, and the Gene column indicates the gene being targeted.

Target	Plant ID	Phenotype	Genotype (allele1 / allele2)	Sequencing type
AtPDS3 coding sequence	gRNA2_HS_116-1	Albino	4bp del / 4bp del	amp-seq
AtPDS3 coding sequence	gRNA2_HS_116-2	Albino	4bp del / 4bp del	amp-seq
AtPDS3 coding sequence	gRNA2_HS_116-3	WT	WT / WT	amp-seq
AtPDS3 coding sequence	gRNA2_HS_116-4	WT	WT / WT	amp-seq
AtPDS3 coding sequence	gRNA2_HS_116-5	WT	4bp del / WT	amp-seq
AtPDS3 coding sequence	gRNA2_HS_116-6	WT	WT / WT	amp-seq
AtPDS3 coding sequence	gRNA2_HS_116-9	WT	WT / WT	amp-seq
AtPDS3 coding sequence	gRNA2_HS_116-10	WT	WT / WT	amp-seq
AtPDS3 coding sequence	gRNA2_HS_116-12	WT	WT / WT	amp-seq
AtPDS3 coding sequence	gRNA2_HS_116-13	WT	WT / WT	amp-seq
AtPDS3 coding sequence	gRNA2_HS_116-14	WT	WT / WT	amp-seq
AtPDS3 coding sequence	gRNA2_HS_116-15	WT	WT / WT	amp-seq
AtPDS3 coding sequence	gRNA2_HS_116-16	WT	WT / WT	amp-seq
AtPDS3 coding sequence	gRNA2_HS_116-17	WT	WT / WT	amp-seq
AtPDS3 coding sequence	gRNA2_HS_116-18	WT	WT / WT	amp-seq
AtPDS3 coding sequence	gRNA2_HS_116-19	WT	WT / WT	amp-seq
AtPDS3 coding sequence	gRNA2_HS_116-20	WT	WT / WT	amp-seq
AtPDS3 coding sequence	gRNA2_HS_116-21	WT	WT / WT	amp-seq
AtPDS3 coding sequence	gRNA2_HS_116-22	WT	WT / WT	amp-seq
AtPDS3 coding sequence	gRNA2_HS_116-23	WT	WT / WT	amp-seq
AtPDS3 coding sequence	gRNA2_HS_116-24	WT	WT / WT	amp-seq
AtPDS3 coding sequence	gRNA2_HS_116-25	WT	WT / WT	amp-seq

AtPDS3 coding sequence	gRNA2_HS_116-26	WT	WT / WT	amp-seq
AtPDS3 coding sequence	gRNA2_HS_116-27	WT	WT / WT	amp-seq
AtPDS3 coding sequence	gRNA2_HS_116-28	WT	4bp del / WT	amp-seq
AtPDS3 coding sequence	gRNA2_HS_116-29	WT	WT / WT	amp-seq
AtPDS3 coding sequence	gRNA2_HS_116-30	Albino	4bp del / 4bp del	amp-seq
AtPDS3 coding sequence	gRNA2_HS_116-31	Albino	4bp del / 4bp del	amp-seq
AtPDS3 coding sequence	gRNA2_HS_116-32	Albino	4bp del / 4bp del	amp-seq
AtPDS3 coding sequence	gRNA2_HS_116-33	Albino	4bp del / 4bp del	amp-seq
AtPDS3 coding sequence	gRNA2_HS_116-34	Albino	4bp del / 4bp del	amp-seq
AtPDS3 coding sequence	gRNA2_HS_116-35	Albino	4bp del / 4bp del	amp-seq
AtPDS3 coding sequence	gRNA2_HS_116-36	Albino	4bp del / 4bp del	amp-seq
AtPDS3 coding sequence	gRNA2_HS_116-38	Albino	4bp del / 4bp del	amp-seq
AtPDS3 coding sequence	gRNA2_HS_116-39	Albino	4bp del / 4bp del	amp-seq
AtPDS3 coding sequence	gRNA2_HS_116-40	Albino	4bp del / 4bp del	amp-seq
AtPDS3 coding sequence	gRNA2_HS_116-41	Albino	4bp del / 4bp del	amp-seq
AtPDS3 coding sequence	gRNA2_HS_116-42	Albino	4bp del / 4bp del	amp-seq
AtPDS3 coding sequence	gRNA2_HS_116-43	Albino	11 bp del / 17bp del	amp-seq
AtPDS3 coding sequence	gRNA2_HS_116-45	Albino	4bp del / 4bp del	amp-seq
AtPDS3 coding sequence	gRNA2_HS_116-46	Albino	4bp del / 4bp del	amp-seq
AtPDS3 coding sequence	gRNA2_HS_116-47	Albino	4bp del / 4bp del	amp-seq
AtPDS3 coding sequence	gRNA2_HS_116-48	Albino	4bp del / 4bp del	amp-seq
AtPDS3 coding sequence	gRNA2_HS_116-49	Albino	10bp del / 12 bp del	amp-seq
AtPDS3 coding sequence	gRNA2_HS_116-50	Albino	4bp del / 4bp del	amp-seq
AtPDS3 coding sequence	gRNA2_HS_116-51	Albino	4bp del / 4bp del	amp-seq

AtPDS3 coding sequence	gRNA2_HS_116-52	Albino	4bp del / 4bp del	amp-seq
AtPDS3 coding sequence	gRNA2_HS_116-53	Albino	4bp del / 4bp del	amp-seq
AtPDS3 coding sequence	gRNA2_HS_116-54	Albino	4bp del / 4bp del	amp-seq
AtPDS3 coding sequence	gRNA2_HS_116-56	Albino	4bp del / 4bp del	amp-seq
AtPDS3 coding sequence	gRNA2_HS_116-57	Albino	4bp del / 4bp del	amp-seq
AtPDS3 coding sequence	gRNA2_HS_116-58	Albino	4bp del / 4bp del	amp-seq
AtPDS3 coding sequence	gRNA2_HS_116-59	Albino	4bp del / 4bp del	amp-seq
AtPDS3 coding sequence	gRNA2_HS_116-60	Albino	4bp del / 4bp del	amp-seq
AtPDS3 coding sequence	gRNA2_HS_116-61	Albino	4bp del / 4bp del	amp-seq
AtPDS3 coding sequence	gRNA2_HS_116-65	Albino	4bp del / 4bp del	amp-seq
AtPDS3 coding sequence	gRNA2_HS_116-72	Albino	4bp del / 4bp del	amp-seq
AtPDS3 coding sequence	gRNA2_HS_116-79	Albino	4bp del / 4bp del	amp-seq
AtPDS3 coding sequence	gRNA2_HS_116-80	Albino	4bp del / 4bp del	amp-seq
AtPDS3 coding sequence	gRNA2_HS_116-84	Albino	4bp del / 4bp del	amp-seq
AtPDS3 coding sequence	gRNA2_HS_116-85	Albino	4bp del / 4bp del	amp-seq
AtPDS3 coding sequence	gRNA2_HS_116-86	Albino	4bp del / 4bp del	amp-seq
AtPDS3 coding sequence	gRNA2_HS_116-90	Albino	17bp del / 11bp del	amp-seq
AtPDS3 coding sequence	gRNA2_HS_116-91	Albino	4bp del / 4bp del	amp-seq
AtPDS3 coding sequence	gRNA2_HS_116-92	Albino	4bp del / 4bp del	amp-seq
AtPDS3 coding sequence	gRNA2_HS_116-94	WT	WT / WT	amp-seq
AtPDS3 coding sequence	gRNA2_HS_116-95	WT	WT / WT	amp-seq
AtPDS3 coding sequence	gRNA2_HS_116-96	WT	WT / WT	amp-seq
AtPDS3 coding sequence	gRNA2_HS_116-97	WT	WT / WT	amp-seq
AtPDS3 coding sequence	gRNA2_HS_116-98	WT	WT / WT	amp-seq

AtPDS3 coding sequence	gRNA2_HS_116-99	WT	WT / WT	amp-seq
AtPDS3 coding sequence	gRNA2_HS_116-100	WT	4bp del / WT	amp-seq
AtPDS3 coding sequence	gRNA2_HS_116-101	WT	WT / WT	amp-seq
AtPDS3 coding sequence	gRNA2_HS_116-102	WT	WT / WT	amp-seq
AtPDS3 coding sequence	gRNA2_HS_116-103	WT	WT / WT	amp-seq
AtPDS3 coding sequence	gRNA2_HS_116-104	WT	WT / WT	amp-seq
AtPDS3 coding sequence	gRNA2_HS_116-105	WT	WT / WT	amp-seq
AtPDS3 coding sequence	gRNA2_HS_116-106	WT	WT / WT	amp-seq
AtPDS3 coding sequence	gRNA2_HS_116-107	WT	WT / WT	amp-seq
AtPDS3 coding sequence	gRNA2_HS_116-108	WT	4bp del / WT	amp-seq
AtPDS3 coding sequence	gRNA2_HS_116-109	WT	WT / WT	amp-seq
AtPDS3 coding sequence	gRNA2_HS_116-110	WT	WT / WT	amp-seq
AtPDS3 coding sequence	gRNA2_HS_116-111	WT	WT / WT	amp-seq
AtPDS3 coding sequence	gRNA2_HS_116-112	WT	WT / WT	amp-seq
AtPDS3 coding sequence	gRNA2_HS_116-113	WT	WT / WT	amp-seq
AtPDS3 coding sequence	gRNA2_HS_116-114	WT	WT / WT	amp-seq
AtPDS3 coding sequence	gRNA2_HS_116-115	WT	WT / WT	amp-seq
AtPDS3 coding sequence	gRNA2_HS_116-116	WT	WT / WT	amp-seq
AtPDS3 coding sequence	gRNA2_HS_116-117	WT	WT / WT	amp-seq
AtPDS3 coding sequence	gRNA2_HS_116-118	WT	WT / WT	amp-seq
AtPDS3 coding sequence	gRNA2_HS_116-119	WT	WT / WT	amp-seq
AtPDS3 coding sequence	gRNA2_HS_116-120	WT	WT / WT	amp-seq
AtPDS3 coding sequence	gRNA2_HS_116-122	WT	WT / WT	amp-seq
AtPDS3 coding sequence	gRNA2_HS_116-123	WT	WT / WT	amp-seq

AtPDS3 coding sequence	gRNA2_HS_116-124	WT	WT / WT	amp-seq
AtPDS3 coding sequence	gRNA2_HS_116-125	WT	WT / WT	amp-seq
AtPDS3 coding sequence	gRNA2_HS_116-126	WT	WT / WT	amp-seq
AtPDS3 coding sequence	gRNA2_HS_116-127	WT	WT / WT	amp-seq
AtPDS3 coding sequence	gRNA2_HS_116-129	WT	WT / WT	amp-seq
AtPDS3 coding sequence	gRNA2_HS_116-132	WT	WT / WT	amp-seq
AtPDS3 coding sequence	gRNA2_HS_116-133	WT	WT / WT	amp-seq
AtPDS3 coding sequence	gRNA2_HS_116-134	WT	WT / WT	amp-seq
AtPDS3 coding sequence	gRNA2_HS_116-135	WT	WT / WT	amp-seq
AtPDS3 coding sequence	gRNA2_HS_116-137	WT	WT / WT	amp-seq
AtPDS3 coding sequence	gRNA2_HS_116-138	WT	WT / WT	amp-seq
AtPDS3 coding sequence	gRNA2_HS_116-140	WT	WT / WT	amp-seq
AtPDS3 coding sequence	gRNA2_HS_116-141	WT	WT / WT	amp-seq
AtPDS3 coding sequence	gRNA2_HS_116-142	WT	WT / WT	amp-seq
AtPDS3 coding sequence	gRNA2_HS_116-143	WT	WT / WT	amp-seq
AtPDS3 coding sequence	gRNA2_HS_116-145	WT	WT / WT	amp-seq
AtPDS3 coding sequence	gRNA2_HS_116-147	WT	WT / WT	amp-seq
AtPDS3 coding sequence	gRNA2_HS_116-148	WT	WT / WT	amp-seq
AtPDS3 coding sequence	gRNA2_HS_116-149	WT	WT / WT	amp-seq
AtPDS3 coding sequence	gRNA2_HS_116-150	WT	WT / WT	amp-seq
AtPDS3 coding sequence	gRNA2_HS_116-151	WT	WT / WT	amp-seq
AtPDS3 coding sequence	gRNA2_HS_116-152	WT	WT / WT	amp-seq
AtPDS3 coding sequence	gRNA2_HS_116-153	WT	WT / WT	amp-seq
AtPDS3 coding sequence	gRNA2_HS_116-154	WT	WT / WT	amp-seq

AtPDS3 coding sequence	gRNA2_HS_116-156	WT	WT / WT	amp-seq
AtPDS3 coding sequence	gRNA2_HS_116-157	WT	WT / WT	amp-seq
AtPDS3 coding sequence	gRNA2_HS_116-158	WT	WT / WT	amp-seq
AtPDS3 coding sequence	gRNA2_HS_116-160	WT	WT / WT	amp-seq
AtPDS3 coding sequence	gRNA2_HS_116-161	WT	WT / WT	amp-seq
AtPDS3 coding sequence	gRNA2_HS_116-162	WT	WT / WT	amp-seq
AtPDS3 coding sequence	gRNA2_HS_116-163	WT	WT / WT	amp-seq
AtPDS3 coding sequence	gRNA2_HS_116-164	WT	WT / WT	amp-seq
AtPDS3 coding sequence	gRNA2_HS_116-165	WT	WT / WT	amp-seq
AtPDS3 coding sequence	gRNA2_HS_116-166	WT	WT / WT	amp-seq
AtPDS3 coding sequence	gRNA2_HS_116-167	WT	WT / WT	amp-seq
AtPDS3 coding sequence	gRNA2_HS_116-168	WT	WT / WT	amp-seq
AtPDS3 coding sequence	gRNA2_HS_116-169	WT	WT / WT	amp-seq
AtPDS3 coding sequence	gRNA2_HS_116-170	WT	WT / WT	amp-seq
AtPDS3 coding sequence	gRNA2_HS_116-171	WT	WT / WT	amp-seq
AtPDS3 coding sequence	gRNA2_HS_116-172	WT	WT / WT	amp-seq
AtPDS3 coding sequence	gRNA2_HS_116-173	WT	WT / WT	amp-seq
AtPDS3 coding sequence	gRNA2_HS_116-174	WT	WT / WT	amp-seq
AtPDS3 coding sequence	gRNA2_HS_116-175	WT	WT / WT	amp-seq
AtPDS3 coding sequence	gRNA2_HS_116-176	WT	WT / WT	amp-seq
AtPDS3 coding sequence	gRNA2_HS_116-177	WT	WT / WT	amp-seq
AtPDS3 coding sequence	gRNA2_HS_116-178	WT	WT / WT	amp-seq
AtPDS3 coding sequence	gRNA2_HS_116-179	WT	WT / WT	amp-seq
AtPDS3 coding sequence	gRNA2_HS_116-180	WT	4bp del / WT	amp-seq

AtPDS3 coding sequence	gRNA2_HS_116-181	WT	WT / WT	amp-seq
AtPDS3 coding sequence	gRNA2_HS_116-182	WT	WT / WT	amp-seq
AtPDS3 coding sequence	gRNA2_HS_116-183	WT	WT / WT	amp-seq
AtPDS3 coding sequence	gRNA2_HS_116-184	WT	WT / WT	amp-seq
AtPDS3 coding sequence	gRNA2_HS_116-185	WT	WT / WT	amp-seq
AtPDS3 coding sequence	gRNA2_HS_116-186	WT	WT / WT	amp-seq
AtPDS3 coding sequence	gRNA2_HS_116-187	WT	WT / WT	amp-seq
AtPDS3 coding sequence	gRNA2_HS_116-188	WT	WT / WT	amp-seq
AtPDS3 coding sequence	gRNA2_HS_116-189	WT	WT / WT	amp-seq
AtPDS3 coding sequence	gRNA2_HS_116-190	WT	WT / WT	amp-seq
AtPDS3 coding sequence	gRNA2_HS_116-191	WT	WT / WT	amp-seq
AtPDS3 coding sequence	gRNA2_HS_116-192	WT	WT / WT	amp-seq
AtPDS3 coding sequence	gRNA2_HS_116-193	WT	WT / WT	amp-seq
AtPDS3 coding sequence	gRNA2_HS_116-194	WT	WT / WT	amp-seq
AtPDS3 coding sequence	gRNA2_HS_116-195	WT	WT / WT	amp-seq
AtPDS3 coding sequence	gRNA2_HS_116-196	WT	WT / WT	amp-seq
AtPDS3 coding sequence	gRNA2_HS_116-198	WT	WT / WT	amp-seq
AtPDS3 coding sequence	gRNA2_HS_116-199	WT	WT / WT	amp-seq
AtPDS3 coding sequence	gRNA2_HS_116-200	WT	WT / WT	amp-seq
AtPDS3 coding sequence	gRNA2_HS_116-201	WT	WT / WT	amp-seq
AtPDS3 coding sequence	gRNA2_HS_116-202	WT	WT / WT	amp-seq
AtPDS3 coding sequence	gRNA2_HS_116-203	WT	WT / WT	amp-seq
AtPDS3 coding sequence	gRNA2_HS_116-204	WT	WT / WT	amp-seq
AtPDS3 coding sequence	gRNA2_HS_116-205	WT	WT / WT	amp-seq

AtPDS3 coding sequence	gRNA2_HS_116-206	WT	WT / WT	amp-seq
AtPDS3 coding sequence	gRNA2_HS_116-207	WT	WT / WT	amp-seq
AtPDS3 coding sequence	gRNA2_HS_116-208	WT	WT / WT	amp-seq
AtPDS3 coding sequence	gRNA2_HS_116-209	WT	WT / WT	amp-seq
AtPDS3 coding sequence	gRNA2_HS_116-210	WT	WT / WT	amp-seq
AtPDS3 coding sequence	gRNA2_HS_116-211	WT	WT / WT	amp-seq
AtPDS3 coding sequence	gRNA2_HS_116-212	WT	WT / WT	amp-seq
AtPDS3 coding sequence	gRNA2_HS_116-213	WT	WT / WT	amp-seq
AtPDS3 coding sequence	gRNA2_HS_116-214	WT	WT / WT	amp-seq
AtPDS3 coding sequence	gRNA2_HS_116-215	WT	4bp del / WT	amp-seq
AtPDS3 coding sequence	gRNA2_HS_116-216	WT	WT / WT	amp-seq
AtPDS3 coding sequence	gRNA2_HS_116-217	WT	4bp del / WT	amp-seq
AtPDS3 coding sequence	gRNA2_HS_116-218	WT	WT / WT	amp-seq
AtPDS3 coding sequence	gRNA2_HS_116-219	WT	WT / WT	amp-seq
AtPDS3 coding sequence	gRNA2_HS_116-220	WT	WT / WT	amp-seq
AtPDS3 coding sequence	gRNA2_HS_116-221	WT	WT / WT	amp-seq
AtPDS3 coding sequence	gRNA2_HS_116-222	WT	WT / WT	amp-seq
AtPDS3 coding sequence	gRNA2_HS_116-223	WT	WT / WT	amp-seq
AtPDS3 coding sequence	gRNA2_HS_116-224	WT	WT / WT	amp-seq
AtPDS3 coding sequence	gRNA2_HS_116-225	WT	WT / WT	amp-seq
AtPDS3 coding sequence	gRNA2_HS_116-226	WT	WT / WT	amp-seq
AtPDS3 coding sequence	gRNA2_HS_116-227	WT	WT / WT	amp-seq
AtPDS3 coding sequence	gRNA2_HS_116-228	WT	WT / WT	amp-seq
AtPDS3 coding sequence	gRNA2_HS_116-229	WT	WT / WT	amp-seq

AtPDS3 coding sequence	gRNA2_HS_116-230	WT	WT / WT	amp-seq
AtPDS3 coding sequence	gRNA2_HS_116-231	WT	WT / WT	amp-seq
AtPDS3 coding sequence	gRNA2_HS_116-232	WT	WT / WT	amp-seq
AtPDS3 coding sequence	gRNA2_HS_116-233	WT	WT / WT	amp-seq
AtPDS3 coding sequence	gRNA2_HS_116-234	WT	WT / WT	amp-seq
AtPDS3 coding sequence	gRNA2_HS_116-235	WT	WT / WT	amp-seq
AtPDS3 coding sequence	gRNA2_HS_116-236	WT	WT / WT	amp-seq
AtPDS3 coding sequence	gRNA2_HS_116-237	WT	WT / WT	amp-seq
AtPDS3 coding sequence	gRNA2_HS_116-238	WT	WT / WT	amp-seq
AtPDS3 coding sequence	gRNA2_HS_116-239	WT	WT / WT	amp-seq
AtPDS3 coding sequence	gRNA2_HS_116-240	WT	WT / WT	amp-seq
AtPDS3 coding sequence	gRNA2_HS_116-241	WT	WT / WT	amp-seq
AtPDS3 coding sequence	gRNA2_HS_116-242	WT	WT / WT	amp-seq
AtPDS3 coding sequence	gRNA2_HS_116-243	WT	WT / WT	amp-seq
AtPDS3 coding sequence	gRNA2_HS_116-244	WT	4bp del / WT	amp-seq
AtPDS3 coding sequence	gRNA2_HS_116-245	WT	WT / WT	amp-seq
AtPDS3 coding sequence	gRNA2_HS_116-246	WT	WT / WT	amp-seq
AtPDS3 coding sequence	gRNA2_HS_116-247	WT	WT / WT	amp-seq
AtPDS3 coding sequence	gRNA2_HS_116-248	WT	WT / WT	amp-seq
AtPDS3 noncoding sequence (promoter)	gRNA12_room_54-35	WT	4bp del / 4bp del	Sanger
AtPDS3 noncoding sequence (promoter)	gRNA12_room_54-36	WT	9bp del / 9bp del	Sanger
AtPDS3 noncoding sequence (promoter)	gRNA12_room_54-37	WT	3bp del / 3bp del	Sanger
AtPDS3 noncoding sequence (promoter)	gRNA12_room_54-40	WT	9bp del / 9bp del	Sanger
AtPDS3 noncoding sequence (promoter)	gRNA12_room_54-42	WT	37bp del / WT	Sanger

AtPDS3 noncoding sequence (promoter)	gRNA12_room_54-43	WT	WT / WT	Sanger
AtPDS3 noncoding sequence (promoter)	gRNA12_room_54-44	WT	WT / WT	Sanger
AtPDS3 noncoding sequence (promoter)	gRNA12_room_54-45	WT	WT / WT	Sanger
AtPDS3 noncoding sequence (promoter)	gRNA12_room_54-46	WT	WT / WT	Sanger
AtPDS3 noncoding sequence (promoter)	gRNA12_room_54-47	WT	WT / WT	Sanger
AtPDS3 noncoding sequence (promoter)	gRNA12_room_54-48	WT	WT / WT	Sanger
AtPDS3 noncoding sequence (promoter)	gRNA12_room_54-49	WT	17bp del / 36bp del	Sanger
AtPDS3 noncoding sequence (promoter)	gRNA12_room_54-50	WT	WT / WT	Sanger
AtPDS3 noncoding sequence (promoter)	gRNA12_room_54-52	WT	WT / WT	Sanger
AtPDS3 noncoding sequence (promoter)	gRNA12_room_54-53	WT	WT / WT	Sanger
AtPDS3 noncoding sequence (promoter)	gRNA12_room_54-54	WT	3bp del / WT	Sanger
AtPDS3 noncoding sequence (promoter)	gRNA12_room_54-55	WT	WT / WT	Sanger
AtPDS3 noncoding sequence (promoter)	gRNA12_room_54-58	WT	17bp del / 36bp del	Sanger
AtPDS3 noncoding sequence (promoter)	gRNA12_room_54-59	WT	9bp del / 9bp del	Sanger
AtPDS3 noncoding sequence (promoter)	gRNA12_room_54-60	WT	WT / WT	Sanger
AtPDS3 noncoding sequence (promoter)	gRNA12_room_54-61	WT	17bp del / 36bp del	Sanger
AtPDS3 noncoding sequence (promoter)	gRNA12_room_54-62	WT	WT / WT	Sanger
AtPDS3 noncoding sequence (promoter)	gRNA12_room_54-63	WT	WT / WT	Sanger
AtPDS3 noncoding sequence (promoter)	gRNA12_room_54-64	WT	WT / WT	Sanger
AtPDS3 noncoding sequence (promoter)	gRNA12_room_54-65	WT	WT / WT	Sanger
AtPDS3 noncoding sequence (promoter)	gRNA12_room_54-66	WT	WT / WT	Sanger
AtPDS3 noncoding sequence (promoter)	gRNA12_room_54-68	WT	12bp del / 12bp del	Sanger
AtPDS3 noncoding sequence (promoter)	gRNA12_room_54-69	WT	3bp del / 3bp del	Sanger
AtPDS3 noncoding sequence (promoter)	gRNA12_room_54-70	WT	WT / WT	Sanger

AtPDS3 noncoding sequence (promoter)	gRNA12_room_54-71	WT	WT / WT	Sanger
AtPDS3 noncoding sequence (promoter)	gRNA12_room_54-72	WT	10bp del / WT	Sanger
AtPDS3 noncoding sequence (promoter)	gRNA12_room_54-73	WT	WT / WT	Sanger
AtPDS3 noncoding sequence (promoter)	gRNA12_room_54-74	WT	WT / WT	Sanger
AtPDS3 noncoding sequence (promoter)	gRNA12_room_54-75	WT	WT / WT	Sanger
AtPDS3 noncoding sequence (promoter)	gRNA12_room_54-76	WT	WT / WT	Sanger
AtPDS3 noncoding sequence (promoter)	gRNA12_room_54-77	WT	WT / WT	Sanger
AtPDS3 noncoding sequence (promoter)	gRNA12_room_54-79	WT	4bp del / WT	Sanger
AtPDS3 noncoding sequence (promoter)	gRNA12_room_54-80	WT	WT / WT	Sanger
AtPDS3 noncoding sequence (promoter)	gRNA12_room_54-84	WT	9bp del / WT	Sanger
AtPDS3 noncoding sequence (promoter)	gRNA12_room_54-86	WT	WT / WT	Sanger
AtPDS3 noncoding sequence (promoter)	gRNA12_room_54-90	WT	WT / WT	Sanger
AtPDS3 noncoding sequence (promoter)	gRNA12_room_54-91	WT	9bp del / 9bp del	Sanger
AtPDS3 noncoding sequence (promoter)	gRNA12_room_54-94	WT	WT / WT	Sanger
AtPDS3 noncoding sequence (promoter)	gRNA12_room_54-95	WT	WT / WT	Sanger
AtPDS3 noncoding sequence (promoter)	gRNA12_room_54-97	WT	3bp del / 3bp del	Sanger
AtPDS3 noncoding sequence (promoter)	gRNA12_room_54-98	WT	WT / WT	Sanger
AtPDS3 noncoding sequence (promoter)	gRNA12_room_54-104	WT	WT / WT	Sanger
AtPDS3 noncoding sequence (promoter)	gRNA12_room_54-105	WT	7bp del / 7bp del	Sanger
AtPDS3 noncoding sequence (promoter)	gRNA12_room_54-106	WT	WT / WT	Sanger
AtPDS3 noncoding sequence (promoter)	gRNA12_room_54-108	WT	WT / WT	Sanger
AtPDS3 noncoding sequence (promoter)	gRNA12_room_54-109	WT	17bp del / 21bp del	Sanger
AtPDS3 noncoding sequence (promoter)	gRNA12_room_54-110	WT	3bp del / WT	Sanger
AtPDS3 noncoding sequence (promoter)	gRNA12_room_54-111	WT	WT / WT	Sanger

AtPDS3 noncoding sequence (promoter)	gRNA12_room_54-112	WT	7bp del / 7bp del	Sanger
AtPDS3 noncoding sequence (promoter)	gRNA12_room_54-113	WT	26bp del / WT	Sanger
AtPDS3 noncoding sequence (promoter)	gRNA12_room_54-114	WT	WT / WT	Sanger
AtPDS3 noncoding sequence (promoter)	gRNA12_room_54-115	WT	WT / WT	Sanger
AtPDS3 noncoding sequence (promoter)	gRNA12_room_54-116	WT	WT / WT	Sanger
AtPDS3 noncoding sequence (promoter)	gRNA12_room_54-117	WT	WT / WT	Sanger
AtPDS3 noncoding sequence (promoter)	gRNA12_room_54-119	WT	WT / WT	Sanger
AtPDS3 noncoding sequence (promoter)	gRNA12_room_54-120	WT	11bp del / 11bp del	Sanger
AtPDS3 noncoding sequence (promoter)	gRNA12_room_54-121	WT	10bp del / WT	Sanger
AtPDS3 noncoding sequence (promoter)	gRNA12_room_54-122	WT	WT / WT	Sanger
AtPDS3 noncoding sequence (promoter)	gRNA12_room_54-123	WT	WT / WT	Sanger
AtPDS3 noncoding sequence (promoter)	gRNA12_room_54-124	WT	10bp del / WT	Sanger
AtPDS3 noncoding sequence (promoter)	gRNA12_room_54-125	WT	WT / WT	Sanger
AtPDS3 noncoding sequence (promoter)	gRNA12_room_54-126	WT	WT / WT	Sanger
AtPDS3 noncoding sequence (promoter)	gRNA12_room_54-127	WT	WT / WT	Sanger
AtPDS3 noncoding sequence (promoter)	gRNA12_room_54-128	WT	4bp del / WT	Sanger
AtPDS3 noncoding sequence (promoter)	gRNA12_room_54-130	WT	38bp del / WT	Sanger
AtPDS3 noncoding sequence (promoter)	gRNA12_room_54-132	WT	WT / WT	Sanger
AtPDS3 noncoding sequence (promoter)	gRNA12_room_54-133	WT	WT / WT	Sanger
AtPDS3 noncoding sequence (promoter)	gRNA12_room_54-134	WT	WT / WT	Sanger
AtPDS3 noncoding sequence (promoter)	gRNA12_room_54-135	WT	WT / WT	Sanger
AtPDS3 noncoding sequence (promoter)	gRNA12_room_54-136	WT	WT / WT	Sanger
AtPDS3 noncoding sequence (promoter)	gRNA12_room_54-138	WT	9bp del / 9bp del	Sanger
AtPDS3 noncoding sequence (promoter)	gRNA12_room_54-139	WT	2bp del / 2bp del	Sanger

AtPDS3 noncoding sequence (promoter)	gRNA12_room_54-140	WT	WT / WT	Sanger
AtPDS3 noncoding sequence (promoter)	gRNA12_room_54-141	WT	WT / WT	Sanger
AtPDS3 noncoding sequence (promoter)	gRNA12_room_54-142	WT	37bp del / WT	Sanger
AtPDS3 noncoding sequence (promoter)	gRNA12_room_54-143	WT	WT / WT	Sanger
AtPDS3 noncoding sequence (promoter)	gRNA12_room_54-144	WT	WT / WT	Sanger
AtPDS3 noncoding sequence (promoter)	gRNA12_room_54-145	WT	WT / WT	Sanger
AtPDS3 noncoding sequence (promoter)	gRNA12_room_54-146	WT	WT / WT	Sanger
AtPDS3 noncoding sequence (promoter)	gRNA12_room_54-148	WT	17bp del / 36bp del	Sanger
AtPDS3 noncoding sequence (promoter)	gRNA12_room_54-149	WT	WT / WT	Sanger
AtPDS3 noncoding sequence (promoter)	gRNA12_room_54-152	WT	WT / WT	Sanger
AtPDS3 noncoding sequence (promoter)	gRNA12_room_54-153	WT	WT / WT	Sanger
AtPDS3 noncoding sequence (promoter)	gRNA12_room_54-154	WT	WT / WT	Sanger
AtPDS3 noncoding sequence (promoter)	gRNA12_room_54-155	WT	WT / WT	Sanger
AtPDS3 noncoding sequence (promoter)	gRNA12_room_54-156	WT	11bp del / 11bp del	Sanger
AtPDS3 noncoding sequence (promoter)	gRNA12_room_54-157	WT	5bp del / WT	Sanger
AtPDS3 noncoding sequence (promoter)	gRNA12_room_54-158	WT	37bp del / WT	Sanger
AtPDS3 noncoding sequence (promoter)	gRNA12_room_54-159	WT	62bp del / 62bp del	Sanger
AtPDS3 noncoding sequence (promoter)	gRNA12_room_54-160	WT	WT / WT	Sanger
AtPDS3 noncoding sequence (promoter)	gRNA12_room_54-161	WT	8bp del / WT	Sanger
AtPDS3 noncoding sequence (promoter)	gRNA12_room_54-162	WT	8bp del / WT	Sanger
AtPDS3 noncoding sequence (promoter)	gRNA12_room_54-163	WT	WT / WT	Sanger
AtPDS3 noncoding sequence (promoter)	gRNA12_room_54-164	WT	4bp del / WT	Sanger
AtPDS3 noncoding sequence (promoter)	gRNA12_room_54-165	WT	WT / WT	Sanger
AtPDS3 noncoding sequence (promoter)	gRNA12_room_54-166	WT	2bp del / WT	Sanger

AtPDS3 noncoding sequence (promoter)	gRNA12_room_54-167	WT	WT / WT	Sanger
AtPDS3 noncoding sequence (promoter)	gRNA12_room_54-168	WT	WT / WT	Sanger
AtPDS3 noncoding sequence (promoter)	gRNA12_room_54-169	WT	WT / WT	Sanger
AtPDS3 noncoding sequence (promoter)	gRNA12_room_54-170	WT	WT / WT	Sanger
AtPDS3 noncoding sequence (promoter)	gRNA12_room_54-171	WT	WT / WT	Sanger
AtPDS3 noncoding sequence (promoter)	gRNA12_room_54-172	WT	WT / WT	Sanger
AtPDS3 noncoding sequence (promoter)	gRNA12_room_54-173	WT	WT / WT	Sanger
AtPDS3 noncoding sequence (promoter)	gRNA12_room_54-175	WT	WT / WT	Sanger
AtPDS3 noncoding sequence (promoter)	gRNA12_room_54-176	WT	WT / WT	Sanger
AtPDS3 noncoding sequence (promoter)	gRNA12_room_54-178	WT	WT / WT	Sanger
AtPDS3 noncoding sequence (promoter)	gRNA12_room_54-179	WT	WT / WT	Sanger
AtPDS3 noncoding sequence (promoter)	gRNA12_room_54-180	WT	WT / WT	Sanger
AtPDS3 noncoding sequence (promoter)	gRNA12_room_54-181	WT	WT / WT	Sanger
AtPDS3 noncoding sequence (promoter)	gRNA12_room_54-182	WT	WT / WT	Sanger
AtPDS3 noncoding sequence (promoter)	gRNA12_room_54-183	WT	WT / WT	Sanger
AtPDS3 noncoding sequence (promoter)	gRNA12_room_54-184	WT	5bp del / WT	Sanger
AtPDS3 noncoding sequence (promoter)	gRNA12_room_54-185	WT	WT / WT	Sanger
AtPDS3 noncoding sequence (promoter)	gRNA12_room_54-186	WT	WT / WT	Sanger
AtPDS3 noncoding sequence (promoter)	gRNA12_room_54-190	WT	9bp del / WT	Sanger
AtPDS3 noncoding sequence (promoter)	gRNA12_room_54-191	WT	15bp del / WT	Sanger
AtPDS3 noncoding sequence (promoter)	gRNA12_room_54-192	WT	WT / WT	Sanger
AtPDS3 noncoding sequence (promoter)	gRNA12_room_54-193	WT	WT / WT	Sanger
AtPDS3 noncoding sequence (promoter)	gRNA12_room_54-194	WT	7bp del / 7bp del	Sanger
AtPDS3 noncoding sequence (promoter)	gRNA12_room_54-195	WT	WT / WT	Sanger

AtPDS3 noncoding sequence (promoter)	gRNA12_room_54-196	WT	WT / WT	Sanger
AtPDS3 noncoding sequence (promoter)	gRNA12_room_54-197	WT	10bp del / 10bp del	Sanger
AtPDS3 noncoding sequence (promoter)	gRNA12_room_54-198	WT	10bp del / WT	Sanger
AtPDS3 noncoding sequence (promoter)	gRNA12_room_54-199	WT	WT / WT	Sanger
AtPDS3 noncoding sequence (promoter)	gRNA12_room_54-200	WT	2bp del / 2bp del	Sanger
AtPDS3 noncoding sequence (promoter)	gRNA12_room_54-202	WT	WT / WT	Sanger
AtPDS3 noncoding sequence (promoter)	gRNA12_room_54-205	WT	WT / WT	Sanger
AtPDS3 noncoding sequence (promoter)	gRNA12_room_54-208	WT	WT / WT	Sanger
AtPDS3 noncoding sequence (promoter)	gRNA12_room_54-209	WT	WT / WT	Sanger
AtPDS3 noncoding sequence (promoter)	gRNA12_room_54-211	WT	WT / WT	Sanger
AtPDS3 noncoding sequence (promoter)	gRNA12_room_54-212	WT	WT / WT	Sanger
AtPDS3 noncoding sequence (promoter)	gRNA12_room_54-213	WT	3bp del / WT	Sanger
AtPDS3 noncoding sequence (promoter)	gRNA12_room_54-214	WT	WT / WT	Sanger
AtPDS3 noncoding sequence (promoter)	gRNA12_room_54-215	WT	4bp del / 4bp del	Sanger
AtPDS3 noncoding sequence (promoter)	gRNA12_room_54-216	WT	WT / WT	Sanger
AtPDS3 noncoding sequence (promoter)	gRNA12_room_54-217	WT	3bp del / WT	Sanger
AtPDS3 noncoding sequence (promoter)	gRNA12_room_54-218	WT	WT / WT	Sanger
AtPDS3 noncoding sequence (promoter)	gRNA12_room_54-219	WT	17bp del / 17bp del	Sanger
AtPDS3 noncoding sequence (promoter)	gRNA12_room_54-220	WT	4bp del / WT	Sanger
AtPDS3 noncoding sequence (promoter)	gRNA12_room_54-221	WT	7bp del / 10bp del	Sanger
AtPDS3 noncoding sequence (promoter)	gRNA12_room_54-222	WT	WT / WT	Sanger
AtPDS3 noncoding sequence (promoter)	gRNA12_room_54-223	WT	WT / WT	Sanger
AtPDS3 noncoding sequence (promoter)	gRNA12_room_54-224	WT	WT / WT	Sanger
AtPDS3 noncoding sequence (promoter)	gRNA12_room_69-2	WT	15bp del / 15bp del	Sanger

AtPDS3 noncoding sequence (promoter)	gRNA12_room_69-4	WT	27bp del / WT	Sanger
AtPDS3 noncoding sequence (promoter)	gRNA12_room_69-5	WT	5bp del / 24bp del	Sanger
AtPDS3 noncoding sequence (promoter)	gRNA12_room_69-7	WT	WT / WT	Sanger
AtPDS3 noncoding sequence (promoter)	gRNA12_room_69-8	WT	7bp del / 15bp del	Sanger
AtPDS3 noncoding sequence (promoter)	gRNA12_room_69-9	WT	1bp del / 1bp del	Sanger
AtPDS3 noncoding sequence (promoter)	gRNA12_room_69-10	WT	WT / WT	Sanger
AtPDS3 noncoding sequence (promoter)	gRNA12_room_69-12	WT	5bp del / 15bp del	Sanger
AtPDS3 noncoding sequence (promoter)	gRNA12_room_69-14	WT	22bp del / 22bp del	Sanger
AtPDS3 noncoding sequence (promoter)	gRNA12_room_69-16	WT	10bp del / 10bp del	Sanger
AtPDS3 noncoding sequence (promoter)	gRNA12_room_69-17	WT	1bp del / 17bp del	Sanger
AtPDS3 noncoding sequence (promoter)	gRNA12_room_69-18	WT	11bp del / WT	Sanger
AtPDS3 noncoding sequence (promoter)	gRNA12_room_69-21	WT	5bp del / 24bp del	Sanger
AtPDS3 noncoding sequence (promoter)	gRNA12_room_69-22	WT	17bp del / WT	Sanger
AtPDS3 noncoding sequence (promoter)	gRNA12_room_69-23	WT	WT / WT	Sanger
AtPDS3 noncoding sequence (promoter)	gRNA12_room_69-28	WT	5bp del / 4bp del	Sanger
AtPDS3 noncoding sequence (promoter)	gRNA12_room_69-30	WT	29bp del / 29bp del	Sanger
AtPDS3 noncoding sequence (promoter)	gRNA12_room_69-33	WT	WT / WT	Sanger
AtPDS3 noncoding sequence (promoter)	gRNA12_room_69-34	WT	5bp del / 5bp del	Sanger
AtPDS3 noncoding sequence (promoter)	gRNA12_room_69-37	WT	WT / WT	Sanger
AtPDS3 noncoding sequence (promoter)	gRNA12_room_69-41	WT	WT / WT	Sanger
AtPDS3 noncoding sequence (promoter)	gRNA12_room_69-44	WT	5bp del / 15bp del	Sanger
AtPDS3 noncoding sequence (promoter)	gRNA12_room_69-51	WT	5bp del / WT	Sanger
AtPDS3 noncoding sequence (promoter)	gRNA12_room_69-53	WT	15bp del / 15bp del	Sanger
AtPDS3 noncoding sequence (promoter)	gRNA12_room_69-55	WT	5bp del / 5bp del	Sanger

AtPDS3 noncoding sequence (promoter)	gRNA12_room_69-59	WT	24bp del / 24bp del	Sanger
AtPDS3 noncoding sequence (promoter)	gRNA12_room_69-63	WT	5bp del / 24bp del	Sanger
AtPDS3 noncoding sequence (promoter)	gRNA12_room_69-66	WT	WT / WT	Sanger
AtPDS3 noncoding sequence (promoter)	gRNA12_room_69-67	WT	WT / WT	Sanger
AtPDS3 noncoding sequence (promoter)	gRNA12_room_69-70	WT	7bp del / WT	Sanger
AtPDS3 noncoding sequence (promoter)	gRNA12_room_69-73	WT	17bp del / WT	Sanger
AtPDS3 noncoding sequence (promoter)	gRNA12_room_69-74	WT	5bp del / 10bp del	Sanger
AtPDS3 noncoding sequence (promoter)	gRNA12_room_69-78	WT	WT / WT	Sanger
AtPDS3 noncoding sequence (promoter)	gRNA12_room_69-80	WT	WT / WT	Sanger
AtPDS3 noncoding sequence (promoter)	gRNA12_room_69-87	WT	13bp del / 14bp del	Sanger
AtPDS3 noncoding sequence (promoter)	gRNA12_room_69-88	WT	11bp del / WT	Sanger
AtPDS3 noncoding sequence (promoter)	gRNA12_room_69-91	WT	WT / WT	Sanger
AtPDS3 noncoding sequence (promoter)	gRNA12_room_69-92	WT	5bp del / 10bp del	Sanger
AtPDS3 noncoding sequence (promoter)	gRNA12_room_69-93	WT	WT / WT	Sanger
AtPDS3 noncoding sequence (promoter)	gRNA12_room_69-94	WT	WT / WT	Sanger
AtPDS3 noncoding sequence (promoter)	gRNA12_room_69-95	WT	3bp del / 23bp del	Sanger
AtPDS3 noncoding sequence (promoter)	gRNA12_room_69-101	WT	7bp del / WT	Sanger
AtPDS3 noncoding sequence (promoter)	gRNA12_room_69-108	WT	WT / WT	Sanger
AtPDS3 noncoding sequence (promoter)	gRNA12_room_69-111	WT	WT / WT	Sanger
AtPDS3 noncoding sequence (promoter)	gRNA12_room_69-115	WT	WT / WT	Sanger
AtPDS3 noncoding sequence (promoter)	gRNA12_room_69-117	WT	10bp del / WT	Sanger
AtPDS3 noncoding sequence (promoter)	gRNA12_room_69-120	WT	17bp del / WT	Sanger
AtPDS3 noncoding sequence (promoter)	gRNA12_room_69-123	WT	WT / WT	Sanger
AtPDS3 noncoding sequence (promoter)	gRNA12_room_69-124	WT	15bp del / 15bp del	Sanger

AtPDS3 noncoding sequence (promoter)	gRNA12_room_69-125	WT	9bp del / 22bp del	Sanger
AtPDS3 noncoding sequence (promoter)	gRNA12_room_69-126	WT	17bp del / 17bp del	Sanger
AtPDS3 noncoding sequence (promoter)	gRNA12_room_69-127	WT	WT / WT	Sanger
AtPDS3 noncoding sequence (promoter)	gRNA12_room_69-129	WT	WT / WT	Sanger
AtPDS3 noncoding sequence (promoter)	gRNA12_room_69-130	WT	WT / WT	Sanger
AtPDS3 noncoding sequence (promoter)	gRNA12_room_69-131	WT	4bp del / 5bp del	Sanger
AtPDS3 noncoding sequence (promoter)	gRNA12_room_69-132	WT	WT / WT	Sanger
AtPDS3 noncoding sequence (promoter)	gRNA12_room_69-134	WT	WT / WT	Sanger
AtPDS3 noncoding sequence (promoter)	gRNA12_room_69-137	WT	4bp del / WT	Sanger
AtPDS3 noncoding sequence (promoter)	gRNA12_room_69-142	WT	15bp del / 5bp del	Sanger
AtPDS3 noncoding sequence (promoter)	gRNA12_room_69-148	WT	WT / WT	Sanger
AtPDS3 noncoding sequence (promoter)	gRNA12_room_69-149	WT	22bp del / 22bp del	Sanger
AtPDS3 noncoding sequence (promoter)	gRNA12_room_69-150	WT	WT / WT	Sanger
AtPDS3 noncoding sequence (promoter)	gRNA12_room_69-151	WT	WT / WT	Sanger
AtPDS3 noncoding sequence (promoter)	gRNA12_room_69-154	WT	11bp del / 11bp del	Sanger
AtPDS3 noncoding sequence (promoter)	gRNA12_room_69-157	WT	WT / WT	Sanger
AtPDS3 noncoding sequence (promoter)	gRNA12_room_69-158	WT	11bp del / WT	Sanger
AtPDS3 noncoding sequence (promoter)	gRNA12_room_69-159	WT	18bp del / WT	Sanger
AtPDS3 noncoding sequence (promoter)	gRNA12_room_69-163	WT	WT / WT	Sanger
AtPDS3 noncoding sequence (promoter)	gRNA12_room_69-170	WT	7bp del / 26bp del	Sanger
AtPDS3 noncoding sequence (promoter)	gRNA12_room_69-171	WT	3bp del / 5bp del	Sanger
AtPDS3 noncoding sequence (promoter)	gRNA12_room_69-172	WT	10bp del / 10bp del	Sanger
AtPDS3 noncoding sequence (promoter)	gRNA12_room_69-175	WT	5bp del / WT	Sanger
AtPDS3 noncoding sequence (promoter)	gRNA12_room_69-183	WT	WT / WT	Sanger

AtPDS3 noncoding sequence (promoter)	gRNA12_room_69-185	WT	5bp del / 5bp del	Sanger
AtPDS3 noncoding sequence (promoter)	gRNA12_room_69-188	WT	1bp del / WT	Sanger
AtCHLI1 coding sequence	gRNA4_2154_12-26_HS_1	Yellow	7bp del / 10bp del	Sanger
AtCHLI1 coding sequence	gRNA4_2154_12-26_HS_2	Yellow	7bp del / 10bp del	Sanger
AtCHLI1 coding sequence	gRNA4_2154_12-26_HS_3	Yellow	6bp del / 6bp del	Sanger
AtCHLI1 coding sequence	gRNA4_2154_12-26_HS_4	Yellow	5bp del / 5bp del	Sanger
AtCHLI1 coding sequence	gRNA4_2154_12-26_HS_5	Yellow	4bp del / 4bp del	Sanger
AtCHLI1 coding sequence	gRNA4_2154_12-26_HS_6	Yellow	4bp del / 4bp del	Sanger
AtCHLI1 coding sequence	gRNA4_2154_12-26_HS_7	Yellow	13bp del / 13bp del	Sanger
AtCHLI1 coding sequence	gRNA4_2154_12-26_HS_8	Yellow	4bp del / 4bp del	Sanger
AtCHLI1 coding sequence	gRNA4_2154_12-26_HS_9	Yellow	43bp del / 43bp del	Sanger
AtCHLI1 coding sequence	gRNA4_2154_12-26_HS_10	Yellow	5bp del / 5bp del	Sanger
AtCHLI1 coding sequence	gRNA4_2154_12-26_HS_11	Yellow	5bp del / 5bp del	Sanger
AtCHLI1 coding sequence	gRNA4_2154_12-26_HS_12	Yellow	5bp del / 5bp del	Sanger
AtCHLI1 coding sequence	gRNA4_2154_12-26_HS_13	Yellow	13bp del / 13bp del	Sanger
AtCHLI1 coding sequence	gRNA4_2154_12-26_HS_14	Yellow	10 bp del / 10 bp del	Sanger
AtCHLI1 coding sequence	gRNA4_2154_12-26_HS_15	WT	WT / WT	Sanger
AtCHLI1 coding sequence	gRNA4_2154_12-26_HS_16	WT	WT / WT	Sanger
AtCHLI1 coding sequence	gRNA4_2154_12-26_HS_17	WT	WT / WT	Sanger
AtCHLI1 coding sequence	gRNA4_2154_12-26_HS_18	WT	WT / WT	Sanger
AtCHLI1 coding sequence	gRNA4_2154_12-26_HS_19	WT	WT / WT	Sanger
AtCHLI1 coding sequence	gRNA4_2154_12-26_HS_20	WT	WT / WT	Sanger
AtCHLI1 coding sequence	gRNA4_2154_12-26_HS_21	Yellow	14bp del / 14bp del	Sanger
AtCHLI1 coding sequence	gRNA4_2154_12-26_HS_22	WT	WT / WT	Sanger

AtCHLI1 coding sequence	gRNA4_2154_12-26_HS_23	WT	WT / WT	Sanger
AtCHLI1 coding sequence	gRNA4_2154_12-26_HS_24	WT	WT / WT	Sanger
AtCHLI1 coding sequence	gRNA4_2154_12-26_HS_25	WT	WT / WT	Sanger
AtCHLI1 coding sequence	gRNA4_2154_12-26_HS_26	WT	WT / WT	Sanger
AtCHLI1 coding sequence	gRNA4_2154_12-26_HS_27	WT	WT / WT	Sanger
AtCHLI1 coding sequence	gRNA4_2154_12-26_HS_28	WT	WT / WT	Sanger
AtCHLI1 coding sequence	gRNA4_2154_12-26_HS_29	WT	WT / WT	Sanger
AtCHLI1 coding sequence	gRNA4_2154_12-26_HS_30	WT	WT / WT	Sanger
AtCHLI1 coding sequence	gRNA4_2154_12-26_HS_31	WT	WT / WT	Sanger
AtCHLI1 coding sequence	gRNA4_2154_12-26_HS_32	WT	WT / WT	Sanger
AtCHLI1 coding sequence	gRNA4_2154_12-26_HS_33	WT	WT / WT	Sanger
AtCHLI1 coding sequence	gRNA4_2154_12-26_HS_34	WT	WT / WT	Sanger
AtCHLI1 coding sequence	gRNA4_2154_12-26_HS_35	WT	4bp del / WT	Sanger
AtCHLI1 coding sequence	gRNA4_2154_12-26_HS_36	WT	WT / WT	Sanger
AtCHLI1 coding sequence	gRNA4_2154_12-26_HS_37	WT	WT / WT	Sanger
AtCHLI1 coding sequence	gRNA4_2154_12-26_HS_38	WT	WT / WT	Sanger
AtCHLI1 coding sequence	gRNA4_2154_12-26_HS_39	WT	WT / WT	Sanger
AtCHLI1 coding sequence	gRNA4_2154_12-26_HS_40	WT	WT / WT	Sanger
AtCHLI1 coding sequence	gRNA4_2154_12-26_HS_41	WT	WT / WT	Sanger
AtCHLI1 coding sequence	gRNA4_2154_12-26_HS_42	WT	WT / WT	Sanger
AtCHLI1 coding sequence	gRNA4_2154_12-26_HS_43	WT	WT / WT	Sanger
AtCHLI1 coding sequence	gRNA4_2154_12-26_HS_44	WT	WT / WT	Sanger
AtCHLI1 coding sequence	gRNA4_2154_12-26_HS_45	WT	WT / WT	Sanger
AtCHLI1 coding sequence	gRNA4_2154_12-26_HS_46	WT	WT / WT	Sanger

AtCHLI1 coding sequence	gRNA4_2154_12-26_HS_47	WT	WT / WT	Sanger
AtCHLI1 coding sequence	gRNA4_2154_12-26_HS_48	WT	WT / WT	Sanger
AtCHLI1 coding sequence	gRNA4_2154_12-26_HS_49	WT	WT / WT	Sanger
AtCHLI1 coding sequence	gRNA4_2154_12-26_HS_50	WT	4bp del / WT	Sanger
AtCHLI1 coding sequence	gRNA4_2154_12-26_HS_51	WT	WT / WT	Sanger
AtCHLI1 coding sequence	gRNA4_2154_12-26_HS_52	WT	WT / WT	Sanger
AtCHLI1 coding sequence	gRNA4_2154_12-26_HS_53	WT	WT / WT	Sanger
AtCHLI1 coding sequence	gRNA4_2154_12-26_HS_54	WT	WT / WT	Sanger
AtCHLI1 coding sequence	gRNA4_2154_12-26_HS_55	WT	WT / WT	Sanger
AtCHLI1 coding sequence	gRNA4_2154_12-26_HS_56	WT	WT / WT	Sanger
AtCHLI1 coding sequence	gRNA4_2154_12-26_HS_57	WT	WT / WT	Sanger
AtCHLI1 coding sequence	gRNA4_2154_12-26_HS_58	WT	WT / WT	Sanger
AtCHLI1 coding sequence	gRNA4_2154_12-26_HS_59	WT	WT / WT	Sanger
AtCHLI1 coding sequence	gRNA4_2154_12-26_HS_60	WT	WT / WT	Sanger
AtCHLI1 coding sequence	gRNA4_2154_12-26_HS_61	WT	WT / WT	Sanger
AtCHLI1 coding sequence	gRNA4_2154_12-26_HS_62	WT	WT / WT	Sanger
AtCHLI1 coding sequence	gRNA4_2154_12-26_HS_63	WT	WT / WT	Sanger
AtCHLI1 coding sequence	gRNA4_2154_12-26_HS_64	WT	WT / WT	Sanger
AtCHLI1 coding sequence	gRNA4_2154_12-26_HS_65	WT	WT / WT	Sanger
AtCHLI1 coding sequence	gRNA4_2154_12-26_HS_66	WT	WT / WT	Sanger
AtCHLI1 coding sequence	gRNA4_2154_12-26_HS_67	WT	WT / WT	Sanger
AtCHLI1 coding sequence	gRNA4_2154_12-26_HS_68	WT	WT / WT	Sanger
AtCHLI1 coding sequence	gRNA4_2154_12-26_HS_69	WT	WT / WT	Sanger
AtCHLI1 coding sequence	gRNA4_2154_12-26_HS_70	WT	WT / WT	Sanger

AtCHLI1 coding sequence	gRNA4_2154_12-26_HS_71	WT	WT / WT	Sanger
AtCHLI1 coding sequence	gRNA4_2154_12-26_HS_72	WT	4bp del / WT	Sanger
AtCHLI1 coding sequence	gRNA4_2154_12-26_HS_73	WT	WT / WT	Sanger
AtCHLI1 coding sequence	gRNA4_2154_12-26_HS_74	WT	WT / WT	Sanger
AtCHLI1 coding sequence	gRNA4_2154_12-26_HS_75	WT	WT / WT	Sanger
AtCHLI1 coding sequence	gRNA4_2154_12-26_HS_76	WT	WT / WT	Sanger
AtCHLI1 coding sequence	gRNA4_2154_12-26_HS_77	WT	WT / WT	Sanger
AtCHLI1 coding sequence	gRNA4_2154_12-26_HS_78	WT	WT / WT	Sanger
AtCHLI1 coding sequence	gRNA4_2154_12-26_HS_79	WT	WT / WT	Sanger
AtCHLI1 coding sequence	gRNA4_2154_12-26_HS_80	WT	WT / WT	Sanger
AtCHLI1 coding sequence	gRNA4_2154_12-26_HS_81	WT	5bp del / WT	Sanger
AtCHLI1 coding sequence	gRNA4_2154_12-26_HS_82	WT	WT / WT	Sanger
AtCHLI1 coding sequence	gRNA4_2154_12-26_HS_83	WT	WT / WT	Sanger
AtCHLI1 coding sequence	gRNA4_2154_12-26_HS_84	WT	WT / WT	Sanger
AtCHLI1 coding sequence	gRNA4_2154_12-26_HS_85	WT	WT / WT	Sanger
AtCHLI1 coding sequence	gRNA4_2154_12-26_HS_86	WT	WT / WT	Sanger
AtCHLI1 coding sequence	gRNA4_2154_12-26_HS_87	WT	WT / WT	Sanger
AtCHLI1 coding sequence	gRNA4_2154_12-26_HS_88	WT	WT / WT	Sanger
AtCHLI1 coding sequence	gRNA4_2154_12-26_HS_89	WT	WT / WT	Sanger
AtCHLI1 coding sequence	gRNA4_2154_12-26_HS_90	WT	WT / WT	Sanger
AtCHLI1 coding sequence	gRNA4_2154_12-26_HS_91	WT	WT / WT	Sanger
AtCHLI1 coding sequence	gRNA4_2154_12-26_HS_92	WT	WT / WT	Sanger
AtCHLI1 coding sequence	gRNA4_2154_12-26_HS_93	WT	WT / WT	Sanger
AtCHLI1 coding sequence	gRNA4_2154_12-26_HS_94	WT	WT / WT	Sanger

AtCHLI1 coding sequence	gRNA4_2154_12-26_HS_95	WT	WT / WT	Sanger
AtCHLI1 coding sequence	gRNA4_2154_12-26_HS_96	WT	WT / WT	Sanger
AtCHLI1 coding sequence	gRNA4_2154_12-26_HS_97	WT	WT / WT	Sanger
AtCHLI1 coding sequence	gRNA4_2154_12-26_HS_98	WT	WT / WT	Sanger
AtCHLI1 coding sequence	gRNA4_2154_12-26_HS_99	WT	WT / WT	Sanger
AtCHLI1 coding sequence	gRNA4_2154_12-26_HS_100	WT	WT / WT	Sanger
AtCHLI1 coding sequence	gRNA4_2154_12-26_HS_101	WT	4bp del / WT	Sanger
AtCHLI1 coding sequence	gRNA4_2154_12-26_HS_102	WT	14bp del / WT	Sanger
AtCHLI1 coding sequence	gRNA4_2154_12-26_HS_103	WT	WT / WT	Sanger
AtCHLI1 coding sequence	gRNA4_2154_12-26_HS_104	WT	WT / WT	Sanger
AtCHLI1 coding sequence	gRNA4_2154_12-26_HS_105	WT	WT / WT	Sanger
AtCHLI1 coding sequence	gRNA4_2154_12-26_HS_106	WT	WT / WT	Sanger
AtCHLI1 coding sequence	gRNA4_2154_12-26_HS_107	WT	WT / WT	Sanger
AtCHLI1 coding sequence	gRNA4_2154_12-26_HS_108	WT	WT / WT	Sanger
AtCHLI1 coding sequence	gRNA4_2154_12-26_HS_109	WT	WT / WT	Sanger
AtCHLI1 coding sequence	gRNA4_2154_12-26_HS_110	WT	WT / WT	Sanger
AtCHLI1 coding sequence	gRNA4_2154_12-26_HS_111	WT	WT / WT	Sanger
AtCHLI1 coding sequence	gRNA4_2154_12-26_HS_112	WT	WT / WT	Sanger
AtCHLI1 coding sequence	gRNA4_2154_12-26_HS_113	WT	4bp del / WT	Sanger
AtCHLI1 coding sequence	gRNA4_2154_12-26_HS_114	WT	WT / WT	Sanger
AtCHLI1 coding sequence	gRNA4_2154_12-26_HS_115	WT	WT / WT	Sanger
AtCHLI1 coding sequence	gRNA4_2154_12-26_HS_116	WT	WT / WT	Sanger
AtCHLI1 coding sequence	gRNA4_2154_12-26_HS_117	WT	WT / WT	Sanger
AtCHLI1 coding sequence	gRNA4_2154_12-26_HS_118	WT	WT / WT	Sanger

AtCHLI1 coding sequence	gRNA4_2154_12-26_HS_119	WT	WT / WT	Sanger
AtCHLI1 coding sequence	gRNA4_2154_12-26_HS_120	WT	WT / WT	Sanger
AtCHLI1 coding sequence	gRNA4_2154_12-26_HS_121	WT	WT / WT	Sanger
AtCHLI1 coding sequence	gRNA4_2154_12-26_HS_122	WT	WT / WT	Sanger
AtCHLI1 coding sequence	gRNA4_2154_12-26_HS_123	WT	4bp del / WT	Sanger
AtCHLI1 coding sequence	gRNA4_2154_12-26_HS_124	WT	WT / WT	Sanger
AtCHLI1 coding sequence	gRNA4_2154_12-26_HS_125	WT	WT / WT	Sanger
AtCHLI1 coding sequence	gRNA4_2154_12-26_HS_126	WT	5bp del / WT	Sanger
AtCHLI1 coding sequence	gRNA4_2154_12-26_HS_127	WT	WT / WT	Sanger
AtCHLI1 coding sequence	gRNA4_2154_12-26_HS_128	WT	WT / WT	Sanger
AtCHLI1 coding sequence	gRNA4_2154_12-26_HS_129	WT	WT / WT	Sanger
AtCHLI1 coding sequence	gRNA4_2154_12-26_HS_130	WT	WT / WT	Sanger
AtCHLI1 coding sequence	gRNA4_2154_12-26_HS_131	WT	WT / WT	Sanger
AtCHLI1 coding sequence	gRNA4_2154_12-26_HS_132	WT	WT / WT	Sanger
AtCHLI1 coding sequence	gRNA4_2154_12-26_HS_133	WT	WT / WT	Sanger
AtCHLI1 coding sequence	gRNA4_2154_12-26_HS_134	WT	WT / WT	Sanger
AtCHLI1 coding sequence	gRNA4_2154_12-26_HS_135	WT	WT / WT	Sanger
AtCHLI1 coding sequence	gRNA4_2154_12-26_HS_136	WT	WT / WT	Sanger
AtCHLI1 coding sequence	gRNA4_2154_12-26_HS_137	WT	WT / WT	Sanger
AtCHLI1 coding sequence	gRNA4_2154_12-26_HS_138	WT	WT / WT	Sanger
AtCHLI1 coding sequence	gRNA4_2154_12-26_HS_139	WT	WT / WT	Sanger
AtCHLI1 coding sequence	gRNA4_2154_12-26_HS_140	WT	WT / WT	Sanger
AtCHLI1 coding sequence	gRNA4_2154_12-26_HS_141	WT	WT / WT	Sanger
AtCHLI1 coding sequence	gRNA4_2154_12-26_HS_142	WT	WT / WT	Sanger

AtCHLI1 coding sequence	gRNA4_2154_12-26_HS_143	WT	WT / WT	Sanger
AtCHLI1 coding sequence	gRNA4_2154_12-26_HS_144	WT	WT / WT	Sanger
AtCHLI1 coding sequence	gRNA4_2154_12-26_HS_145	WT	WT / WT	Sanger
AtCHLI1 coding sequence	gRNA4_2154_12-26_HS_146	WT	WT / WT	Sanger
AtCHLI1 coding sequence	gRNA4_2154_12-26_HS_147	WT	WT / WT	Sanger
AtCHLI1 coding sequence	gRNA4_2154_12-26_HS_148	WT	WT / WT	Sanger
AtCHLI1 coding sequence	gRNA4_2154_12-26_HS_149	WT	WT / WT	Sanger
AtCHLI1 coding sequence	gRNA4_2154_12-26_HS_150	WT	WT / WT	Sanger
AtCHLI1 coding sequence	gRNA4_2154_12-26_HS_151	WT	WT / WT	Sanger
AtCHLI1 coding sequence	gRNA4_2154_12-26_HS_152	WT	WT / WT	Sanger
AtCHLI1 coding sequence	gRNA4_2154_12-26_HS_153	WT	WT / WT	Sanger
AtCHLI1 coding sequence	gRNA4_2154_12-26_HS_154	WT	WT / WT	Sanger
AtCHLI1 coding sequence	gRNA4_2154_12-26_HS_155	WT	WT / WT	Sanger
AtCHLI1 coding sequence	gRNA4_2154_12-26_HS_156	WT	WT / WT	Sanger
AtCHLI1 coding sequence	gRNA4_2154_12-26_HS_157	WT	WT / WT	Sanger
AtCHLI1 coding sequence	gRNA4_2154_12-26_HS_158	WT	WT / WT	Sanger
AtCHLI1 coding sequence	gRNA6_2156_12-65_1	Yellow	3bp del / 3bp del	Sanger
AtCHLI1 coding sequence	gRNA6_2156_12-65_2	WT	WT / WT	Sanger
AtCHLI1 coding sequence	gRNA6_2156_12-65_3	WT	WT / WT	Sanger
AtCHLI1 coding sequence	gRNA6_2156_12-65_4	WT	WT / WT	Sanger
AtCHLI1 coding sequence	gRNA6_2156_12-65_5	WT	WT / WT	Sanger
AtCHLI1 coding sequence	gRNA6_2156_12-65_6	WT	WT / WT	Sanger
AtCHLI1 coding sequence	gRNA6_2156_12-65_7	WT	WT / WT	Sanger
AtCHLI1 coding sequence	gRNA6_2156_12-65_8	WT	WT / WT	Sanger

AtCHLI1 coding sequence	gRNA6_2156_12-65_9	WT	WT / WT	Sanger
AtCHLI1 coding sequence	gRNA6_2156_12-65_10	WT	WT / WT	Sanger
AtCHLI1 coding sequence	gRNA6_2156_12-65_11	WT	WT / WT	Sanger
AtCHLI1 coding sequence	gRNA6_2156_12-65_12	WT	WT / WT	Sanger
AtCHLI1 coding sequence	gRNA6_2156_12-65_13	WT	WT / WT	Sanger
AtCHLI1 coding sequence	gRNA6_2156_12-65_14	WT	WT / WT	Sanger
AtCHLI1 coding sequence	gRNA6_2156_12-65_15	WT	WT / WT	Sanger
AtCHLI1 coding sequence	gRNA6_2156_12-65_16	WT	WT / WT	Sanger
AtCHLI1 coding sequence	gRNA6_2156_12-65_17	WT	WT / WT	Sanger
AtCHLI1 coding sequence	gRNA6_2156_12-65_18	WT	WT / WT	Sanger
AtCHLI1 coding sequence	gRNA6_2156_12-65_19	WT	WT / WT	Sanger
AtCHLI1 coding sequence	gRNA6_2156_12-65_20	WT	WT / WT	Sanger
AtCHLI1 coding sequence	gRNA6_2156_12-65_21	WT	WT / WT	Sanger
AtCHLI1 coding sequence	gRNA6_2156_12-65_22	WT	WT / WT	Sanger
AtCHLI1 coding sequence	gRNA6_2156_12-65_23	WT	WT / WT	Sanger
AtCHLI1 coding sequence	gRNA6_2156_12-65_24	WT	WT / WT	Sanger
AtCHLI1 coding sequence	gRNA6_2156_12-65_25	WT	WT / WT	Sanger
AtCHLI1 coding sequence	gRNA6_2156_12-65_26	WT	WT / WT	Sanger
AtCHLI1 coding sequence	gRNA6_2156_12-65_27	WT	WT / WT	Sanger
AtCHLI1 coding sequence	gRNA6_2156_12-65_28	WT	WT / WT	Sanger
AtCHLI1 coding sequence	gRNA6_2156_12-65_29	WT	WT / WT	Sanger
AtCHLI1 coding sequence	gRNA6_2156_12-65_30	WT	WT / WT	Sanger
AtCHLI1 coding sequence	gRNA6_2156_12-65_31	WT	WT / WT	Sanger
AtCHLI1 coding sequence	gRNA6_2156_12-65_32	WT	WT / WT	Sanger

AtCHLI1 coding sequence	gRNA6_2156_12-65_33	WT	WT / WT	Sanger
AtCHLI1 coding sequence	gRNA6_2156_12-65_34	WT	WT / WT	Sanger
AtCHLI1 coding sequence	gRNA6_2156_12-65_35	WT	WT / WT	Sanger
AtCHLI1 coding sequence	gRNA6_2156_12-65_36	WT	WT / WT	Sanger
AtCHLI1 coding sequence	gRNA6_2156_12-65_37	WT	WT / WT	Sanger
AtCHLI1 coding sequence	gRNA6_2156_12-65_38	WT	WT / WT	Sanger
AtCHLI1 coding sequence	gRNA6_2156_12-65_39	WT	WT / WT	Sanger
AtCHLI1 coding sequence	gRNA6_2156_12-65_40	WT	WT / WT	Sanger
AtCHLI1 coding sequence	gRNA6_2156_12-65_41	WT	WT / WT	Sanger
AtCHLI1 coding sequence	gRNA6_2156_12-65_42	WT	WT / WT	Sanger
AtCHLI1 coding sequence	gRNA6_2156_12-65_43	WT	WT / WT	Sanger
AtCHLI1 coding sequence	gRNA6_2156_12-65_44	WT	WT / WT	Sanger
AtCHLI1 coding sequence	gRNA6_2156_12-65_45	WT	WT / WT	Sanger
AtCHLI1 coding sequence	gRNA6_2156_12-65_46	WT	WT / WT	Sanger
AtCHLI1 coding sequence	gRNA6_2156_12-65_47	WT	WT / WT	Sanger
AtCHLI1 coding sequence	gRNA6_2156_12-65_48	WT	WT / WT	Sanger
AtCHLI1 coding sequence	gRNA6_2156_12-65_49	WT	WT / WT	Sanger
AtCHLI1 coding sequence	gRNA6_2156_12-65_50	WT	WT / WT	Sanger
AtCHLI1 coding sequence	gRNA6_2156_12-65_51	WT	WT / WT	Sanger
AtCHLI1 coding sequence	gRNA6_2156_12-65_52	WT	WT / WT	Sanger
AtCHLI1 coding sequence	gRNA6_2156_12-65_53	WT	WT / WT	Sanger
AtCHLI1 coding sequence	gRNA6_2156_12-65_54	WT	WT / WT	Sanger
AtCHLI1 coding sequence	gRNA6_2156_12-65_55	WT	WT / WT	Sanger
AtCHLI1 coding sequence	gRNA6_2156_12-65_56	WT	WT / WT	Sanger

AtCHLI1 coding sequence	gRNA6_2156_12-65_57	WT	WT / WT	Sanger
AtCHLI1 coding sequence	gRNA6_2156_12-65_58	WT	WT / WT	Sanger
AtCHLI1 coding sequence	gRNA6_2156_12-65_59	WT	WT / WT	Sanger
AtCHLI1 coding sequence	gRNA6_2156_12-65_60	WT	WT / WT	Sanger
AtCHLI1 coding sequence	gRNA6_2156_12-65_61	WT	WT / WT	Sanger
AtCHLI1 coding sequence	gRNA6_2156_12-65_62	WT	WT / WT	Sanger
AtCHLI1 coding sequence	gRNA6_2156_12-65_63	WT	WT / WT	Sanger
AtCHLI1 coding sequence	gRNA6_2156_12-65_64	WT	WT / WT	Sanger
AtCHLI1 coding sequence	gRNA6_2156_12-65_65	WT	WT / WT	Sanger
AtCHLI1 coding sequence	gRNA6_2156_12-65_66	WT	WT / WT	Sanger
AtCHLI1 coding sequence	gRNA6_2156_12-65_67	WT	WT / WT	Sanger
AtCHLI1 coding sequence	gRNA6_2156_12-65_68	WT	WT / WT	Sanger
AtCHLI1 coding sequence	gRNA6_2156_12-65_69	WT	WT / WT	Sanger
AtCHLI1 coding sequence	gRNA6_2156_12-65_70	WT	WT / WT	Sanger
AtCHLI1 coding sequence	gRNA6_2156_12-65_71	WT	WT / WT	Sanger
AtCHLI1 coding sequence	gRNA6_2156_12-65_73	Yellow	3bp del / 3bp del	Sanger
AtCHLI1 coding sequence	gRNA6_2156_12-65_74	WT	WT / WT	Sanger
AtCHLI1 coding sequence	gRNA6_2156_12-65_75	WT	WT / WT	Sanger
AtCHLI1 coding sequence	gRNA6_2156_12-65_76	WT	WT / WT	Sanger
AtCHLI1 coding sequence	gRNA6_2156_12-65_77	WT	WT / WT	Sanger
AtCHLI1 coding sequence	gRNA6_2156_12-65_78	WT	WT / WT	Sanger
AtCHLI1 coding sequence	gRNA6_2156_12-65_79	WT	WT / WT	Sanger
AtCHLI1 coding sequence	gRNA6_2156_12-65_80	WT	WT / WT	Sanger
AtCHLI1 coding sequence	gRNA6_2156_12-65_81	WT	WT / WT	Sanger

AtCHLI1 coding sequence	gRNA6_2156_12-65_82	WT	WT / WT	Sanger
AtCHLI1 coding sequence	gRNA6_2156_12-65_83	WT	WT / WT	Sanger
AtCHLI1 coding sequence	gRNA6_2156_12-65_84	WT	WT / WT	Sanger
AtCHLI1 coding sequence	gRNA6_2156_12-65_85	WT	WT / WT	Sanger
AtCHLI1 coding sequence	gRNA6_2156_12-65_86	WT	WT / WT	Sanger
AtCHLI1 coding sequence	gRNA6_2156_12-65_87	WT	WT / WT	Sanger
AtCHLI1 coding sequence	gRNA6_2156_12-65_88	WT	WT / WT	Sanger
AtCHLI1 coding sequence	gRNA6_2156_12-65_89	WT	WT / WT	Sanger
AtCHLI1 coding sequence	gRNA6_2156_12-65_90	WT	WT / WT	Sanger
AtCHLI1 coding sequence	gRNA6_2156_12-65_91	WT	WT / WT	Sanger
AtCHLI1 coding sequence	gRNA6_2156_12-65_92	WT	WT / WT	Sanger
AtCHLI1 coding sequence	gRNA6_2156_12-65_93	WT	WT / WT	Sanger
AtCHLI1 coding sequence	gRNA6_2156_12-65_94	WT	WT / WT	Sanger
AtCHLI1 coding sequence	gRNA6_2156_12-65_95	WT	WT / WT	Sanger
AtCHLI1 coding sequence	gRNA6_2156_12-65_96	WT	WT / WT	Sanger
AtCHLI1 coding sequence	gRNA6_2156_12-65_97	WT	WT / WT	Sanger
AtCHLI1 coding sequence	gRNA6_2156_12-65_98	WT	WT / WT	Sanger
AtCHLI1 coding sequence	gRNA6_2156_12-65_99	WT	WT / WT	Sanger
AtCHLI1 coding sequence	gRNA6_2156_12-65_100	WT	WT / WT	Sanger
AtCHLI1 coding sequence	gRNA6_2156_12-65_101	WT	WT / WT	Sanger
AtCHLI1 coding sequence	gRNA6_2156_12-65_103	WT	WT / WT	Sanger
AtCHLI1 coding sequence	gRNA6_2156_12-65_104	WT	WT / WT	Sanger
AtCHLI1 coding sequence	gRNA6_2156_12-65_105	WT	WT / WT	Sanger
AtCHLI1 coding sequence	gRNA6_2156_12-65_106	WT	3bp del / WT	Sanger

AtCHLI1 coding sequence	gRNA6_2156_12-65_107	WT	WT / WT	Sanger
AtCHLI1 coding sequence	gRNA6_2156_12-65_108	WT	WT / WT	Sanger
AtCHLI1 coding sequence	gRNA6_2156_12-65_109	WT	WT / WT	Sanger
AtCHLI1 coding sequence	gRNA6_2156_12-65_110	WT	WT / WT	Sanger
AtCHLI1 coding sequence	gRNA6_2156_12-65_111	WT	WT / WT	Sanger
AtCHLI1 coding sequence	gRNA6_2156_12-65_113	WT	WT / WT	Sanger
AtCHLI1 coding sequence	gRNA6_2156_12-65_115	WT	WT / WT	Sanger
AtCHLI1 coding sequence	gRNA6_2156_12-65_116	WT	WT / WT	Sanger
AtCHLI1 coding sequence	gRNA6_2156_12-65_118	WT	WT / WT	Sanger
AtCHLI1 coding sequence	gRNA6_2156_12-65_119	WT	WT / WT	Sanger
AtCHLI1 coding sequence	gRNA6_2156_12-65_120	WT	WT / WT	Sanger
AtCHLI1 coding sequence	gRNA6_2156_12-65_121	WT	WT / WT	Sanger
AtCHLI1 coding sequence	gRNA6_2156_12-65_122	WT	WT / WT	Sanger
AtCHLI1 coding sequence	gRNA6_2156_12-65_123	WT	WT / WT	Sanger
AtCHLI1 coding sequence	gRNA6_2156_12-65_124	WT	WT / WT	Sanger
AtCHLI1 coding sequence	gRNA6_2156_12-65_125	WT	WT / WT	Sanger
AtCHLI1 coding sequence	gRNA6_2156_12-65_126	WT	WT / WT	Sanger
AtCHLI1 coding sequence	gRNA6_2156_12-65_127	WT	WT / WT	Sanger
AtCHLI1 coding sequence	gRNA6_2156_12-65_128	WT	WT / WT	Sanger
AtCHLI1 coding sequence	gRNA6_2156_12-65_129	WT	WT / WT	Sanger
AtCHLI1 coding sequence	gRNA6_2156_12-65_130	WT	WT / WT	Sanger
AtCHLI1 coding sequence	gRNA6_2156_12-65_131	WT	WT / WT	Sanger
AtCHLI1 coding sequence	gRNA6_2156_12-65_132	WT	WT / WT	Sanger
AtCHLI1 coding sequence	gRNA6_2156_12-65_133	WT	WT / WT	Sanger

AtCHLl1 coding sequence	gRNA6_2156_12-65_134	WT	WT / WT	Sanger
AtCHLl1 coding sequence	gRNA6_2156_12-65_135	WT	WT / WT	Sanger
AtCHLl1 coding sequence	gRNA6_2156_12-65_136	WT	WT / WT	Sanger
AtCHLl1 coding sequence	gRNA6_2156_12-65_137	WT	WT / WT	Sanger
AtCHLl1 coding sequence	gRNA6_2156_12-65_139	WT	WT / WT	Sanger
AtCHLl1 coding sequence	gRNA6_2156_12-65_140	WT	WT / WT	Sanger
AtCHLl1 coding sequence	gRNA6_2156_12-65_141	WT	WT / WT	Sanger
AtCHLl1 coding sequence	gRNA6_2156_12-65_142	WT	WT / WT	Sanger
AtCHLl1 coding sequence	gRNA6_2156_12-65_143	WT	WT / WT	Sanger
AtCHLl1 coding sequence	gRNA6_2156_12-65_144	WT	WT / WT	Sanger
AtCHLl1 coding sequence	gRNA6_2156_12-65_145	WT	WT / WT	Sanger

Supplementary Table 2: Phenotype and genotype of the progenies from TRV-infected plants. The Target column lists the gene and genomic context of each gRNA. The Plant ID column indicates an internal identifier for each plant sampled. The Phenotype column indicates whether the plant was WT (green), yellow, or albino. The Genotype (allele1 / allele2) column lists the alleles of the *AtPDS3* or *AtCHLl1* gene determined by amp-seq or Sanger Sequencing. The Sequencing type column indicates if amp-seq of Sanger sequencing was used. WT is an abbreviation for wild type, bp is an abbreviation for base pair, and del is an abbreviation for deletion.

Sample	Reads count	Unmapped reads count	Mapping rate (%)	Coverage
WT-rep1	331996121	2122433	99.36	817.69
WT-rep2	314298698	1969352	99.37	774.20
gRNA2_HS_116-2	315840117	1628611	99.48	778.87
gRNA2_HS_116-32	357227432	2397598	99.33	879.55
gRNA2_HS_116-43	265745567	1688428	99.36	654.55

Supplementary Table 3: Whole genome sequencing read count and mapping rate of WT control and albino mutant plants.

Sample	GATK	Strealk2	GATK+Strealk2	Filter with WT	Filter by depth > 30	Manually check
WT	40852	24406	NA	NA	NA	NA
albino-plant2	36346	20659	16296	28	25	5
albino-plant32	36342	20991	16629	35	30	5
albino-plant43	36989	21928	17111	80	71	4

Supplementary Table 4: WGS variant detection of WT control and albino mutant plants using GATK and Strelka2.

DNA	Chromosome	Location	Direction	Mismatches	Variants
TTcATtAGGCAAATTCaCtGC	chr1	1799061	+	4	No SNP/Indel
TTGATAAGGaAgATTCGtCtC	chr1	21688156	+	4	No SNP/Indel
TTGATAAGcCAtATTCtCCcC	chr1	28196564	+	4	No SNP/Indel
TTGATcAGGaAAATTCGCgGa	chr1	29172329	-	4	No SNP/Indel
TTGATcAGGCAAATTCGgaGC	chr2	2183162	+	3	No SNP/Indel
aaGcTAAGGCAAATcCGCCGC	chr2	10574134	-	4	No SNP/Indel
TTGATAAGaacAtTTCGCCGC	chr2	11679602	-	4	No SNP/Indel
gTcATAAGtCAcATTCGCCGC	chr2	12173059	+	4	No SNP/Indel
TTGATAAGtCAAAgTCtaCGC	chr2	13554186	-	4	No SNP/Indel
TTGATActGCAAAgTCGCCtC	chr2	16402169	+	4	No SNP/Indel
TacAgAAGGCAAATTCcCCGC	chr3	381121	-	4	No SNP/Indel
TTGATAAtGCAAcTTCtCCGt	chr3	527026	-	4	No SNP/Indel
TTcATcAGGCAtATTCGCCGt	chr3	13200296	-	4	No SNP/Indel
TTGgTAAGGCAAATTCttaGC	chr4	8560337	+	4	No SNP/Indel
gTGAaAAcGCAAATTCGCgGC	chr5	3918356	+	4	No SNP/Indel
TTGATAAGtCAAAaTCcCCaC	chr5	10937249	-	4	No SNP/Indel
TTGATAAtGCAAATTacCCGt	chr5	18593314	-	4	No SNP/Indel
TTGATAAGGCAAtcTCtaCGC	chr5	26138716	+	4	No SNP/Indel

Supplementary Table 5: Off-target editing analysis using Cas-OFFinder. The DNA column contains the sequence of the potential off-target site, with lower case letters indicating a mismatch compared to the actual target site. The Chromosome, Location, and Direction columns indicate where in the *Arabidopsis* genome that potential off-target site is located. The mismatch column indicates the number of mismatches in the potential off-target site relative to

the actual target site sequence. The Variants column lists the off-target editing result for that site, with every off-target site analyzed as wild type (No SNP/Indel).

Plasmid	Description
pMK003	Cloning vector for PaqCI Golden Gate assembly of ISDra2 guides into ISDra2 plant expression vector
pMK025	Cloning vector for PaqCI Golden Gate assembly of ISYmu1 guides into ISYmu1 plant expression vector
pMK024	Cloning vector for PaqCI Golden Gate assembly of ISAam1 guides into ISAam1 plant expression vector
pMK026	ISDra2 g12 cloned into pMK003
pMK027	ISDra2 g13 cloned into pMK003
pMK028	ISDra2 g8 cloned into pMK003
pMK029	ISDra2 g14 cloned into pMK003
pMK030	ISDra2 g4 cloned into pMK003
pMK031	ISDra2 g15 cloned into pMK003
pMK032	ISDra2 g17 cloned into pMK003
pMK033	ISDra2 g18 cloned into pMK003
pMK034	ISDra2 g5 cloned into pMK003
pMK035	ISDra2 g20 cloned into pMK003
pMK036	ISDra2 g22 cloned into pMK003
pMK037	ISDra2 g1 cloned into pMK003
pMK038	ISDra2 g2 cloned into pMK003
pMK039	ISDra2 g3 cloned into pMK003
pMK040	ISDra2 g16 cloned into pMK003

pMK041	ISDra2 g19 cloned into pMK003
pMK042	ISDra2 g9 cloned into pMK003
pMK043	ISDra2 g21 cloned into pMK003
pMK044	ISDra2 g11 cloned into pMK003
pMK045	ISDra2 g7 cloned into pMK003
pMK060	ISYmu1 g1 cloned into pMK025
pMK061	ISYmu1 g2 cloned into pMK025
pMK062	ISYmu1 g3 cloned into pMK025
pMK063	ISYmu1 g4 cloned into pMK025
pMK064	ISYmu1 g5 cloned into pMK025
pMK065	ISYmu1 g7 cloned into pMK025
pMK066	ISYmu1 g8 cloned into pMK025
pMK067	ISYmu1 g9 cloned into pMK025
pMK068	ISYmu1 g10 cloned into pMK025
pMK070	ISYmu1 g12 cloned into pMK025
pMK050	ISAam1 g11 cloned into pMK024
pMK051	ISAam1 g12 cloned into pMK024
pMK052	ISAam1 g13 cloned into pMK024
pMK053	ISAam1 g14 cloned into pMK024
pMK054	ISAam1 g15 cloned into pMK024
pMK055	ISAam1 g16 cloned into pMK024
pMK056	ISAam1 g17 cloned into pMK024
pTW2065	ISYmu1 PDS3 gRNA2 TRV2 Architecture_A

pTW2066	ISYmu1 PDS3 gRNA2 TRV2 Architecture_B
pTW2082	ISYmu1 PDS3 gRNA12 TRV2 Architecture_B
pTW2153	ISYmu1 CHLI1 gRNA3 TRV2 Architecture_B
pTW2154	ISYmu1 CHLI1 gRNA4 TRV2 Architecture_B
pTW2155	ISYmu1 CHLI1 gRNA5 TRV2 Architecture_B
pTW2156	ISYmu1 CHLI1 gRNA6 TRV2 Architecture_B
pTW2157	ISYmu1 CHLI1 gRNA7 TRV2 Architecture_B
pTW2159	ISYmu1 CHLI1 gRNA9 TRV2 Architecture_B
pMK435	Cloning vector for PaqCI Golden Gate assembly of ISYmu1 gRNAs into TRV2

Supplementary Table 6: Plasmids used in this study. The Plasmid column lists the name of each plasmid used. The Description column provides a TnpB and gRNA description of each plasmid used, along with the intermediate plant expression vector that was used to create it.

Oligo name	Oligo sequence	Oligo description
MK_29476	TCAAgcgttggagcatataacaga	cloning ISDra2 gRNA_20_bp_gRNA_12_Top
Trevor_28675	GGCCtctgttatatgctccaacgc	cloning ISDra2 gRNA_20_bp_gRNA_12_Bottom
MK_29477	TCAAttacgaattgatgaccatat	cloning ISDra2 gRNA_20_bp_gRNA_1_Top
Trevor_28677	GGCCatatggtcatcaattcgtaa	cloning ISDra2 gRNA_20_bp_gRNA_1_Bottom
MK_29478	TCAAaaggcaaattcgccgcagaa	cloning ISDra2 gRNA_20_bp_gRNA_2_Top
Trevor_28679	GGCCttctgcggcgaatttgcctt	cloning ISDra2 gRNA_20_bp_gRNA_2_Bottom
MK_29479	TCAAtaaagagaggaaattgcagg	cloning ISDra2 gRNA_20_bp_gRNA_13_Top
Trevor_28681	GGCCcctgcaatttcctctcttta	cloning ISDra2 gRNA_20_bp_gRNA_13_Bottom
MK_29480	TCAAtggtctcactttccgaatta	cloning ISDra2 gRNA_20_bp_gRNA_8_Top
Trevor_28683	GGCCtaattcggaaagtgagacca	cloning ISDra2 gRNA_20_bp_gRNA_8_Bottom
MK_29481	TCAAtcacattaagcctagaaact	cloning ISDra2 gRNA_20_bp_gRNA_3_Top
Trevor_28685	GGCCagtttctaggcttaatgtga	cloning ISDra2 gRNA_20_bp_gRNA_3_Bottom
MK_29482	TCAAggtagagctgataagatata	cloning ISDra2 gRNA_20_bp_gRNA_14_Top
Trevor_28687	GGCCtatatcttatcagctctacc	cloning ISDra2 gRNA_20_bp_gRNA_14_Bottom
MK_29483	TCAAcccaagttctccaaataaat	cloning ISDra2 gRNA_20_bp_gRNA_4_Top
Trevor_28689	GGCCatttatttggagaacttggg	cloning ISDra2 gRNA_20_bp_gRNA_4_Bottom
MK_29484	TCAAaaaattggattaatgtgcac	cloning ISDra2 gRNA_20_bp_gRNA_15_Top
Trevor_28691	GGCCgtgcacattaatccaatttt	cloning ISDra2 gRNA_20_bp_gRNA_15_Bottom
MK_29485	TCAAcaatacaaataaatacatgc	cloning ISDra2 gRNA_20_bp_gRNA_17_Top
Trevor_28693	GGCCgcatgtatttatttgtattg	cloning ISDra2 gRNA_20_bp_gRNA_17_Bottom
MK_29486	TCAAcaattcaagctaattataga	cloning ISDra2 gRNA_20_bp_gRNA_18_Top
Trevor_28695	GGCCtctataattagcttgaattg	cloning ISDra2 gRNA_20_bp_gRNA_18_Bottom
MK_29487	TCAAtactattaaatgtcaaaatc	cloning ISDra2 gRNA_20_bp_gRNA_16_Top

Trevor_28697	GGCCgattttgacatttaatagta	cloning ISDra2 gRNA_20_bp_gRNA_16_Bottom
MK_29488	TCAAtacccatcctaaagtatggg	cloning ISDra2 gRNA_20_bp_gRNA_5_Top
Trevor_28699	GGCCcccatactttaggatgggta	cloning ISDra2 gRNA_20_bp_gRNA_5_Bottom
MK_29489	TCAAgagcttaacttggtagagta	cloning ISDra2 gRNA_20_bp_gRNA_19_Top
Trevor_28701	GGCCtactctaccaagttaagctc	cloning ISDra2 gRNA_20_bp_gRNA_19_Bottom
MK_29490	TCAAttgtcagctttcttatggat	cloning ISDra2 gRNA_20_bp_gRNA_20_Top
Trevor_28703	GGCCatccataagaaagctgacaa	cloning ISDra2 gRNA_20_bp_gRNA_20_Bottom
MK_29491	TCAAagctttgaaccggtttcttc	cloning ISDra2 gRNA_20_bp_gRNA_9_Top
Trevor_28705	GGCCgaagaaaccggttcaaagct	cloning ISDra2 gRNA_20_bp_gRNA_9_Bottom
MK_29492	TCAAgttgattaacttgtactacc	cloning ISDra2 gRNA_20_bp_gRNA_21_Top
Trevor_28707	GGCCggtagtacaagttaatcaac	cloning ISDra2 gRNA_20_bp_gRNA_21_Bottom
MK_29493	TCAAtaacttgtactacctcatcc	cloning ISDra2 gRNA_20_bp_gRNA_22_Top
Trevor_28709	GGCCggatgaggtagtacaagtta	cloning ISDra2 gRNA_20_bp_gRNA_22_Bottom
MK_29494	TCAAcgaaaactgaagaacacata	cloning ISDra2 gRNA_20_bp_gRNA_11_Top
Trevor_28711	GGCCtatgtgttcttcagttttcg	cloning ISDra2 gRNA_20_bp_gRNA_11_Bottom
MK_29495	TCAAaaattcaacatctttctcta	cloning ISDra2 gRNA_20_bp_gRNA_7_Top
Trevor_28713	GGCCtagagaaagatgttgaattt	cloning ISDra2 gRNA_20_bp_gRNA_7_Bottom
MK_29758	TCAAttacgaattgatgacc	cloning ISYmu1 gRNA_16_bp_gRNA_1_Top
MK_29759	GGCCggtcatcaattcgtaa	cloning ISYmu1 gRNA_16_bp_gRNA_1_Bottom
MK_29760	TCAAaaggcaaattcgccgc	cloning ISYmu1 gRNA_16_bp_gRNA_2_Top
MK_29761	GGCCgcggcgaatttgcctt	cloning ISYmu1 gRNA_16_bp_gRNA_2_Bottom
MK_29762	TCAAtcacattaagcctaga	cloning ISYmu1 gRNA_16_bp_gRNA_3_Top
MK_29763	GGCCtctaggcttaatgtga	cloning ISYmu1 gRNA_16_bp_gRNA_3_Bottom
MK_29764	TCAAcccaagttctccaaat	cloning ISYmu1 gRNA_16_bp_gRNA_4_Top

MK_29765	GGCCatttggagaacttggg	cloning ISYmu1 gRNA_16_bp_gRNA_4_Bottom
MK_29766	TCAAtacccatcctaaagta	cloning ISYmu1 gRNA_16_bp_gRNA_5_Top
MK_29767	GGCCtactttaggatgggta	cloning ISYmu1 gRNA_16_bp_gRNA_5_Bottom
MK_29770	TCAAaaattcaacatctttc	cloning ISYmu1 gRNA_16_bp_gRNA_7_Top
MK_29771	GGCCgaaagatgttgaattt	cloning ISYmu1 gRNA_16_bp_gRNA_7_Bottom
MK_29772	TCAAtggtctcactttccga	cloning ISYmu1 gRNA_16_bp_gRNA_8_Top
MK_29773	GGCCtcggaaagtgagacca	cloning ISYmu1 gRNA_16_bp_gRNA_8_Bottom
MK_29774	TCAAagctttgaaccggttt	cloning ISYmu1 gRNA_16_bp_gRNA_9_Top
MK_29775	GGCCaaaccggttcaaagct	cloning ISYmu1 gRNA_16_bp_gRNA_9_Bottom
MK_29776	TCAAtaacttgtactacctc	cloning ISYmu1 gRNA_16_bp_gRNA_10_Top
MK_29777	GGCCgaggtagtacaagtta	cloning ISYmu1 gRNA_16_bp_gRNA_10_Bottom
MK_29780	TCAAgcgttggagcatataa	cloning ISYmu1 gRNA_16_bp_gRNA_12_Top
MK_29781	GGCCttatatgctccaacgc	cloning ISYmu1 gRNA_16_bp_gRNA_12_Bottom
MK_29818	TCACcaattcatctggtatc	cloning ISAam1 gRNA_16_bp_gRNA_11_Top
MK_29819	GGCCgataccagatgaattg	cloning ISAam1 gRNA_16_bp_gRNA_11_Bottom
MK_29820	TCACccaagaacaagcctta	cloning ISAam1 gRNA_16_bp_gRNA_12_Top
MK_29821	GGCCtaaggcttgttcttgg	cloning ISAam1 gRNA_16_bp_gRNA_12_Bottom
MK_29822	TCACgttttgtcctcttctc	cloning ISAam1 gRNA_16_bp_gRNA_13_Top
MK_29823	GGCCgagaagaggacaaaac	cloning ISAam1 gRNA_16_bp_gRNA_13_Bottom
MK_29824	TCACcaatttacctatctta	cloning ISAam1 gRNA_16_bp_gRNA_14_Top
MK_29825	GGCCtaagataggtaaattg	cloning ISAam1 gRNA_16_bp_gRNA_14_Bottom
MK_29826	TCACcacataattgaaaaga	cloning ISAam1 gRNA_16_bp_gRNA_15_Top
MK_29827	GGCCtcttttcaattatgtg	cloning ISAam1 gRNA_16_bp_gRNA_15_Bottom
MK_29828	TCACaatttgttacacaact	cloning ISAam1 gRNA_16_bp_gRNA_16_Top

MK_29829	GGCCagttgtgtaacaaatt	cloning ISAam1 gRNA_16_bp_gRNA_16_Bottom
MK_29830	TCACtttgtgtggtatttaa	cloning ISAam1 gRNA_16_bp_gRNA_17_Top
MK_29831	GGCCttaaataccacacaaa	cloning ISAam1 gRNA_16_bp_gRNA_17_Bottom

Supplementary Table 7: Oligos used for cloning gRNA sequences into intermediate TnpB plant expression vectors. The Oligo name column is an internal name for each oligo used. The Oligo sequence column lists the sequence of each oligo used for cloning the gRNA. The Oligo description column provides details for cloning each TnpB gRNA target site.

Plasmid	PCR template	Oligo 1	Oligo 2	Reaction #
pTW2065	pDK3888	gaatttcctttaccattgacgtcagtgtcgttggtagcat	tgtaatccatCTCGTTAACTCGGGTAAGTGATACA	1
pTW2065	pMK061	AGTTAACGAGatggattacaaggatgatgatgataagga	CCAACTGAGCTACGGgcggcgaatttgccttTTGA	2
pTW2065	pDK3888	aggcaaattcgccgcCCGTAGCTCAGTTGGTTAGAGC	TGGTCACCTGTAATTCACACGTGGTGGTGGTGGTGGT	3
pTW2066	pDK3888	gaatttcctttaccattgacgtcagtgtcgttggtagcat	tgtaatccatCTCGTTAACTCGGGTAAGTGATACA	1
pTW2066	pMK061	AGTTAACGAGatggattacaaggatgatgatgataagga	TCTAACCAACTGAGCTACGGgtcccattcgccatgcc	2
pTW2066	pDK3888	catggcgaatgggacCCGTAGCTCAGTTGGTTAGAGC	TGGTCACCTGTAATTCACACGTGGTGGTGGTGGTGGT	3
pTW2082	pDK3888	gaatttcctttaccattgacgtcagtgtcgttggtagcat	tgtaatccatCTCGTTAACTCGGGTAAGTGATACA	1
pTW2082	pMK070	AGTTAACGAGatggattacaaggatgatgatgataagga	TCTAACCAACTGAGCTACGGgtcccattcgccatgcc	2
pTW2082	pDK3888	catggcgaatgggacCCGTAGCTCAGTTGGTTAGAGC	TGGTCACCTGTAATTCACACGTGGTGGTGGTGGTGGT	3
pTW2153	N/A	tcaaCCAAAGATTGGTGGTG	ggccCACCACCAATCTTTGG	N/A
pTW2154	N/A	tcaaCTGTTACCTGAGATTA	ggccTAATCTCAGGTAACAG	N/A
pTW2155	N/A	tcaaCTTCCTTTGGGTGCAA	ggccTTGCACCCAAAGGAAG	N/A
pTW2156	N/A	tcaaGAAGTTAATCTCTTGG	ggccCCAAGAGATTAACTTC	N/A
pTW2157	N/A	tcaaGTTCTTTTGGATTCAG	ggccCTGAATCCAAAAGAAC	N/A
pTW2159	N/A	tcaaCGGTTTGGTATGCATG	ggccCATGCATACCAAACCG	N/A

Supplementary Table 8: Cloning TRV Cargo Architecture_A and Architecture_B plasmids. The Plasmid column lists the name of each plasmid created. The PCR template column lists the name of each plasmid used for PCR DNA template. The Oligo 1 and Oligo 2 columns provide the sequence used to amplify fragments using the corresponding PCR template. The reaction column indicates the individual reaction for each PCR. The three PCR reactions, along with the restriction enzyme digested pDK3888, were used for NEB Hifi assembly to create the TRV2 vector listed in the Plasmid column.

Gene	TnpB	Guide	F primer	R primer	Sequencing type
AtPDS3	ISDra2	gRNA1	ACACTCTTTCCCTACACGACGCTCTTCCGATCTaccaaattacgttgagatgcatggtc	GTGACTGGAGTTCAGACGTGTGCTCTTCCGATCTgctcttttgttgcttacgaaacatgt	amp-seq
AtPDS3	ISDra2	gRNA2	ACACTCTTTCCCTACACGACGCTCTTCCGATCTgaagcagttgtgagttaagttggaga	GTGACTGGAGTTCAGACGTGTGCTCTTCCGATCTttgtcttaagcgcttgagaagtgg	amp-seq
AtPDS3	ISDra2	gRNA3	ACACTCTTTCCCTACACGACGCTCTTCCGATCTatgtcttcagtcttgtgctacacacc	GTGACTGGAGTTCAGACGTGTGCTCTTCCGATCTaaacggaacaaacaagaacctaacct	amp-seq
AtPDS3	ISDra2	gRNA4	ACACTCTTTCCCTACACGACGCTCTTCCGATCTtctcttcatgttcttgttggaagtcgg	GTGACTGGAGTTCAGACGTGTGCTCTTCCGATCTgtgctggtaggacatctgggaagt	amp-seq
AtPDS3	ISDra2	gRNA5	ACACTCTTTCCCTACACGACGCTCTTCCGATCTgacgtcaggaagaacatggtcatttg	GTGACTGGAGTTCAGACGTGTGCTCTTCCGATCTagaacatttcagcgctaatgctacaa	amp-seq
AtPDS3	ISDra2	gRNA7	ACACTCTTTCCCTACACGACGCTCTTCCGATCTtagcttcaaatgagggaccaggctt	GTGACTGGAGTTCAGACGTGTGCTCTTCCGATCTtgtagaggacgacatggttcacagtt	amp-seq
AtPDS3	ISDra2	gRNA8	ACACTCTTTCCCTACACGACGCTCTTCCGATCTcacaaacctctgttgcttgaagc	GTGACTGGAGTTCAGACGTGTGCTCTTCCGATCTcattgggtacctaggagctgtaac	amp-seq
AtPDS3	ISDra2	gRNA9	ACACTCTTTCCCTACACGACGCTCTTCCGATCTgcgctaaactttataaaccctgatg	GTGACTGGAGTTCAGACGTGTGCTCTTCCGATCTtatgattgccagtgagataaagattc	amp-seq
AtPDS3	ISDra2	gRNA11	ACACTCTTTCCCTACACGACGCTCTTCCGATCTgaatcgagattacatgcaggtctta	GTGACTGGAGTTCAGACGTGTGCTCTTCCGATCTccaacactgtatccaactaagtattc	amp-seq
AtPDS3	ISDra2	gRNA12	ACACTCTTTCCCTACACGACGCTCTTCCGATCTgaaccgacccgagaagagatttg	GTGACTGGAGTTCAGACGTGTGCTCTTCCGATCTtggaatacacacatttgtacaacca	amp-seq
AtPDS3	ISDra2	gRNA13	ACACTCTTTCCCTACACGACGCTCTTCCGATCTtggagttctcgaaatttcatctagtg	GTGACTGGAGTTCAGACGTGTGCTCTTCCGATCTcggaaggatgcagataaactagca	amp-seq
AtPDS3	ISDra2	gRNA14	ACACTCTTTCCCTACACGACGCTCTTCCGATCTtctgtaagtccaaaactcataccct	GTGACTGGAGTTCAGACGTGTGCTCTTCCGATCTcttccaacaagaacatgaagagagt	amp-seq
AtPDS3	ISDra2	gRNA15	ACACTCTTTCCCTACACGACGCTCTTCCGATCTacagtggttgaactattagttagtgg	GTGACTGGAGTTCAGACGTGTGCTCTTCCGATCTcaaaatctataattagcttgaattgatcaatac	amp-seq
AtPDS3	ISDra2	gRNA16	ACACTCTTTCCCTACACGACGCTCTTCCGATCTgtgaggttagtaactatgatgcatgt	GTGACTGGAGTTCAGACGTGTGCTCTTCCGATCTaagacaaatgaccatgttcttcctg	amp-seq
AtPDS3	ISDra2	gRNA17	GTGACTGGAGTTCAGACGTGTGCTCTTCCGATCTacagtggttgaactattagttagtgg	ACACTCTTTCCCTACACGACGCTCTTCCGATCTcctcaaacattcatcagttctttgat	amp-seq
AtPDS3	ISDra2	gRNA18	GTGACTGGAGTTCAGACGTGTGCTCTTCCGATCTacagtggttgaactattagttagtgg	ACACTCTTTCCCTACACGACGCTCTTCCGATCTcctcaaacattcatcagttctttgat	amp-seq

AtPDS3	ISDra2	gRNA19	ACACTCTTTCCCTACACGACGCTCTTCCGATCTaagtaggctattgagttcacagttt	GTGACTGGAGTTCAGACGTGTGCTCTTCCGATCTatgaaaccacaacaaccacatg	amp-seq
AtPDS3	ISDra2	gRNA20	ACACTCTTTCCCTACACGACGCTCTTCCGATCTcttggtagagtagttagcatgctga	GTGACTGGAGTTCAGACGTGTGCTCTTCCGATCTccctggacaagtatacaattaatcag	amp-seq
AtPDS3	ISDra2	gRNA21	ACACTCTTTCCCTACACGACGCTCTTCCGATCTtgttgcagcttatgacacacaataag	GTGACTGGAGTTCAGACGTGTGCTCTTCCGATCTaatctttatctcactggcaatcatag	amp-seq
AtPDS3	ISDra2	gRNA22	ACACTCTTTCCCTACACGACGCTCTTCCGATCTtgttgcagcttatgacacacaataag	GTGACTGGAGTTCAGACGTGTGCTCTTCCGATCTaatctttatctcactggcaatcatag	amp-seq
AtPDS3	ISYmu1	gRNA1	ACACTCTTTCCCTACACGACGCTCTTCCGATCTaccaaattacgttgagatgcatggtc	GTGACTGGAGTTCAGACGTGTGCTCTTCCGATCTgctcttttgttgcttacgaaacatgt	amp-seq
AtPDS3	ISYmu1	gRNA2	ACACTCTTTCCCTACACGACGCTCTTCCGATCTgaagcagttgtgagttaagttggaga	GTGACTGGAGTTCAGACGTGTGCTCTTCCGATCTttgtcttaagcgcttgagaagtgg	amp-seq
AtPDS3	ISYmu1	gRNA3	ACACTCTTTCCCTACACGACGCTCTTCCGATCTatgtcttcagtcttgtgctacacacc	GTGACTGGAGTTCAGACGTGTGCTCTTCCGATCTaaacggaacaaacaagaacctaacct	amp-seq
AtPDS3	ISYmu1	gRNA4	ACACTCTTTCCCTACACGACGCTCTTCCGATCTtctcttcatgttcttgttggaagtcgg	GTGACTGGAGTTCAGACGTGTGCTCTTCCGATCTgtgctggtaggacatctgggaagt	amp-seq
AtPDS3	ISYmu1	gRNA5	ACACTCTTTCCCTACACGACGCTCTTCCGATCTgacgtcaggaagaacatggtcatttg	GTGACTGGAGTTCAGACGTGTGCTCTTCCGATCTagaacatttcagcgctaatgctacaa	amp-seq
AtPDS3	ISYmu1	gRNA7	ACACTCTTTCCCTACACGACGCTCTTCCGATCTtagcttcaaatgagggaccaggctt	GTGACTGGAGTTCAGACGTGTGCTCTTCCGATCTaagtacttctgttttgtgtaatctcc	amp-seq
AtPDS3	ISYmu1	gRNA8	ACACTCTTTCCCTACACGACGCTCTTCCGATCTcacaaacctctgttgcttgaagc	GTGACTGGAGTTCAGACGTGTGCTCTTCCGATCTcattgggtacctaggagctgtaac	amp-seq
AtPDS3	ISYmu1	gRNA9	ACACTCTTTCCCTACACGACGCTCTTCCGATCTgcgctaaactttataaaccctgatg	GTGACTGGAGTTCAGACGTGTGCTCTTCCGATCTtatgattgccagtgagataaagattc	amp-seq
AtPDS3	ISYmu1	gRNA10	GTGACTGGAGTTCAGACGTGTGCTCTTCCGATCTgaatctttatctcactggcaatcata	ACACTCTTTCCCTACACGACGCTCTTCCGATCTtgttgcagcttatgacacacaataag	amp-seq
AtPDS3	ISYmu1	gRNA12	ACACTCTTTCCCTACACGACGCTCTTCCGATCTgaaccgacccgagaagagatttg	GTGACTGGAGTTCAGACGTGTGCTCTTCCGATCTtggaatacacacatttgtacaacca	amp-seq
AtPDS3	ISAam1	gRNA11	GTGACTGGAGTTCAGACGTGTGCTCTTCCGATCTagtttcttactcactaatggaagcac	ACACTCTTTCCCTACACGACGCTCTTCCGATCTaaggagcttcaggatatcgactacaa	amp-seq
AtPDS3	ISAam1	gRNA12	GTGACTGGAGTTCAGACGTGTGCTCTTCCGATCTgcaggtgaatttcttatttatgcagt	ACACTCTTTCCCTACACGACGCTCTTCCGATCTgaaggttacttctgcaaaaaaacaaca	amp-seq
AtPDS3	ISAam1	gRNA13	ACACTCTTTCCCTACACGACGCTCTTCCGATCTcgtctggaccaagaaagttgt	GTGACTGGAGTTCAGACGTGTGCTCTTCCGATCTcagaaacgtgagatgtcaaatctgt	amp-seq
AtPDS3	ISAam1	gRNA14	ACACTCTTTCCCTACACGACGCTCTTCCGATCTtgttaagttgttattgtgttctctgg	GTGACTGGAGTTCAGACGTGTGCTCTTCCGATCTgaaaatatgtaaaccagtctcatacc	amp-seq

AtPDS3	ISAam1	gRNA15	GTGACTGGAGTTCAGACGTGTGCTCTTCCGATCTcgctgcgtctcctgtttctctac	ACACTCTTTCCCTACACGACGCTCTTCCGATCTccggtggaagcaaatcagac	amp-seq
AtPDS3	ISAam1	gRNA16	ACACTCTTTCCCTACACGACGCTCTTCCGATCTtgcaaggaggaggagtatttgg	GTGACTGGAGTTCAGACGTGTGCTCTTCCGATCTtatccgattccgcttagtttgc	amp-seq
AtPDS3	ISAam1	gRNA17	GTGACTGGAGTTCAGACGTGTGCTCTTCCGATCTaccaaattacgttgagatgcatggtc	ACACTCTTTCCCTACACGACGCTCTTCCGATCTtggtcttgtttggttttcagacc	amp-seq
AtPDS3	ISYmu1	gRNA12	tgttacacaacttattatgattgggct	ggaagtagccgataacaaaatggag	Sanger
AtCHLI1	ISYmu1	gRNA4	ACACTCTTTCCCTACACGACGCTCTTCCGATCTGTGGTGTTATGATTATGGGAGATAGAG	GTGACTGGAGTTCAGACGTGTGCTCTTCCGATCTTCGCAATAACAGGAACTTGCTC	amp-seq
AtCHLI1	ISYmu1	gRNA6	ACACTCTTTCCCTACACGACGCTCTTCCGATCTAAGCCTTTGAGCCTGGTTTG	GTGACTGGAGTTCAGACGTGTGCTCTTCCGATCTCGGGTGAGAAATCGAAATCCC	amp-seq
AtCHLI1	ISYmu1	gRNA9	ACACTCTTTCCCTACACGACGCTCTTCCGATCTGCGAGGTTTATCTTGATCGGTTC	GTGACTGGAGTTCAGACGTGTGCTCTTCCGATCTTCACGGAAATCCTTTGGGTTACTA	amp-seq
AtCHLI1	ISYmu1	gRNA4	ACACTCTTTCCCTACACGACGCTCTTCCGATCTgaagttaggaacatgattgttgttggt	GTGACTGGAGTTCAGACGTGTGCTCTTCCGATCTTCACGGAAATCCTTTGGGTTACTA	Sanger
AtCHLI1	ISYmu1	gRNA6	ACACTCTTTCCCTACACGACGCTCTTCCGATCTgaagttaggaacatgattgttgttggt	GTGACTGGAGTTCAGACGTGTGCTCTTCCGATCTTCACGGAAATCCTTTGGGTTACTA	Sanger

Supplementary Table 9: Primers used for amp-seq and Sanger sequencing. The TnpB and Guide columns indicate the site being targeted for each TnpB. The F primer and R primer columns list the oligo sequences used to amplify genomic DNA for amp-seq or Sanger Sequencing.

oligo name	Oligo sequence	Description
SP9238	TGTATAGACTGTTTGAGATCGGCG	RT-PCR primer to check for the presence/absence of TRV in mutant albino plants
SP9239	GTAATAACGCTTACGTAGGCGAGG	RT-PCR primer to check for the presence/absence of TRV in mutant albino plants
IPP2 RT-PCR Fw	GTATGAGTTGCTTCTCCAGCAAAG	RNA control for RT-PCR
IPP2 RT-PCR Rev	GAGGATGGCTGCAACAAGTGT	RNA control for RT-PCR

Supplementary Table 10: Oligos for RT-PCR. The name of each oligo is listed in the Oligo name column. Each oligo sequence is provided in the Oligo sequence column, with a description of what the oligos were used for in the Description column.
