# Supplementary material for: Viral delivery of an RNA-guided genome editor for transgene-free germline editing in Arabidopsis
Source: Nat Plants. 2025 Apr 22;11(5):967–76. doi: 10.1038/s41477-025-01989-9 (PMC12095077; doi:10.1038/s41477-025-01989-9)
Supplement: Supplementary file 2 — Reporting Summary [file 41477_2025_1989_MOESM2_ESM.pdf]

nature portfolio

Corresponding author(s): Steven Jacobsen

Last updated by author(s): March 13, 2025

Reporting Summary

Nature Portfolio wishes to improve the reproducibility of the work that we publish. This form provides structure for consistency and transparency in reporting. For further information on Nature Portfolio policies, see our Editorial Policies and the Editorial Policy Checklist.

Statistics

For all statistical analyses, confirm that the following items are present in the figure legend, table legend, main text, or Methods section.

n/a	Confirmed	
☐	☒	The exact sample size (*n*) for each experimental group/condition, given as a discrete number and unit of measurement
☒	☐	A statement on whether measurements were taken from distinct samples or whether the same sample was measured repeatedly
☒	☐	The statistical test(s) used AND whether they are one- or two-sided<br>*Only common tests should be described solely by name; describe more complex techniques in the Methods section.*
☒	☐	A description of all covariates tested
☒	☐	A description of any assumptions or corrections, such as tests of normality and adjustment for multiple comparisons
☒	☐	A full description of the statistical parameters including central tendency (e.g. means) or other basic estimates (e.g. regression coefficient) AND variation (e.g. standard deviation) or associated estimates of uncertainty (e.g. confidence intervals)
☒	☐	For null hypothesis testing, the test statistic (e.g. *F*, *t*, *r*) with confidence intervals, effect sizes, degrees of freedom and *P* value noted<br>*Give P values as exact values whenever suitable.*
☒	☐	For Bayesian analysis, information on the choice of priors and Markov chain Monte Carlo settings
☒	☐	For hierarchical and complex designs, identification of the appropriate level for tests and full reporting of outcomes
☒	☐	Estimates of effect sizes (e.g. Cohen's *d*, Pearson's *r*), indicating how they were calculated

Our web collection on statistics for biologists contains articles on many of the points above.

Software and code

Policy information about availability of computer code

Data collection	No software was used for data collection.
Data analysis	All code used in this manuscript was from published manuscripts and cited in the text. NGS amp-seq reads were trimmed using Trim Galore default setting. The remaining reads were mapped to the target genomeregion using BWA aligner (v0.7.17, BWA-MEM algorithm). sorted and indexted bam files were used as input for further analysis by the CrispRvariants R package (v1.14.0). For off target analysis, whole genome sequencing reads were aligned to TAIR10 reference genome using BWA-mem (v0.7.17) with default parameter. GATK (4.2.0.0) MarkDuplicateSpark was used to remove PCR duplicate reads. Then, GATK HaplotypeCaller was used to call raw variants. GATK, (Strelka (v2.9.2), and BedTools (v2.26.0) were used for SNP/InDel calling.

For manuscripts utilizing custom algorithms or software that are central to the research but not yet described in published literature, software must be made available to editors and reviewers. We strongly encourage code deposition in a community repository (e.g. GitHub). See the Nature Portfolio guidelines for submitting code & software for further information.

Data

Policy information about availability of data

All manuscripts must include a data availability statement. This statement should provide the following information, where applicable:

- Accession codes, unique identifiers, or web links for publicly available datasets
- A description of any restrictions on data availability
- For clinical datasets or third party data, please ensure that the statement adheres to our policy

All the amp-seq data generated in this study will be accessible at NCBI Sequence Read Archive under BioProject PRJNA1124592. Whole genome sequencing data is accessible at BioProject PRJNA1146711

Research involving human participants, their data, or biological material

Policy information about studies with human participants or human data. See also policy information about sex, gender (identity/presentation), and sexual orientation and race, ethnicity and racism.

Reporting on sex and gender	N.A.
Reporting on race, ethnicity, or other socially relevant groupings	N.A.
Population characteristics	N.A.
Recruitment	N.A.
Ethics oversight	N.A.

Note that full information on the approval of the study protocol must also be provided in the manuscript.

Field-specific reporting

Please select the one below that is the best fit for your research. If you are not sure, read the appropriate sections before making your selection.

☒ Life sciences ☐ Behavioural & social sciences ☐ Ecological, evolutionary & environmental sciences

For a reference copy of the document with all sections, see nature.com/documents/nr-reporting-summary-flat.pdf

Life sciences study design

All studies must disclose on these points even when the disclosure is negative.

Sample size	No sample size calculation was performed. For the bacterial interference assay and protoplast experiments we tested at least two replicates (usually three) per treatment to allow for comparison between replicates.For protoplast experiments with two replicates, one of the replicates failed to amplify for NGS. For the transgenic editing experiments we analyzed data from all of the transgenic plants we were able to create using the floral dip method. For the TRV delivery experiments we did not predetermine any sample sizes. The trasngenic and TRV plant samples sizes were determined by the amount of available greenhouse space.
Data exclusions	No data was excluded from this study.
Replication	To verify reproducibility of experiments, biological replicates were performed for each experiment.
Randomization	All samples were randomly collected from the subject materials, except where noted in the methods section. For example, when collecting tissue to quantify somatic editing in plants that underwent TRV delivery, three random leaves distal to the delivery site were collected. However, if white or yellow sectors of cells were present on leaves distal to the TRV delivery site, tissue from those leaves was collected.
Blinding	The investigators were blinded to group allocation during data collection and/or analysis for all experiments.

Reporting for specific materials, systems and methods

We require information from authors about some types of materials, experimental systems and methods used in many studies. Here, indicate whether each material, system or method listed is relevant to your study. If you are not sure if a list item applies to your research, read the appropriate section before selecting a response.

Materials & experimental systems

n/a	Involved in the study	
☒	☐	Antibodies
☒	☐	Eukaryotic cell lines
☒	☐	Palaeontology and archaeology
☒	☐	Animals and other organisms
☒	☐	Clinical data
☒	☐	Dual use research of concern
☐	☒	Plants

Methods

n/a	Involved in the study	
☒	☐	ChIP-seq
☒	☐	Flow cytometry
☒	☐	MRI-based neuroimaging

Dual use research of concern

Policy information about dual use research of concern

Hazards

Could the accidental, deliberate or reckless misuse of agents or technologies generated in the work, or the application of information presented in the manuscript, pose a threat to:

No	Yes	
☒	☐	Public health
☒	☐	National security
☒	☐	Crops and/or livestock
☒	☐	Ecosystems
☒	☐	Any other significant area

Experiments of concern

Does the work involve any of these experiments of concern:

No	Yes	
☒	☐	Demonstrate how to render a vaccine ineffective
☒	☐	Confer resistance to therapeutically useful antibiotics or antiviral agents
☒	☐	Enhance the virulence of a pathogen or render a nonpathogen virulent
☒	☐	Increase transmissibility of a pathogen
☒	☐	Alter the host range of a pathogen
☒	☐	Enable evasion of diagnostic/detection modalities
☒	☐	Enable the weaponization of a biological agent or toxin
☒	☐	Any other potentially harmful combination of experiments and agents

Plants

Seed stocks

The ku70 SALK T-DNA mutant was used in this study. It can be obtained from the Arabidopsis Biological Resources Center (ABRC) with the stock number SALK_123114. the rdr6 mutant genotype was previously generated in the Jacobsen lab using CRISPR-Cas9.

Novel plant genotypes

The Arabidopsis transgenic lines were generated using the T-DNA-mediated floral dip transformation method. Transgenic T1 seeds were grown on hygromycin selection plates to identify transgenic plants. The transgenic plants expressed the ISYmu1 TnpB targeting gRNA site 2 (aaggcaaattcgccgc) or gRNA site 12 (gcgttggagcatataa). 61 WT ISYmu1 gRNA2 plants were created (42 room temp and 19 heat shock), 11 rdr6 ISYmu1 gRNA2 plants were created (8 room temp and 3 heat shock), 33 WT ISYmu1 gRNA12 plants were created (12 room temp and 21 heat shock), 65 rdr6 ISYmu1 gRNA12 plants were created (41 room temp and 24 heat shock).

For the TRV delivery experiments, the agroflood method was used to deliver the TnpB and gRNA to the Arabidopsis plants. We analyzed data from 18 ku70 samples using PDS3 gRNA2 TRV2 Architecture_A with the heat shock treatment, 8 ku70 samples using PDS3 gRNA2 TRV2 Architecture_A room temperature treatment, 12 ku70 samples using PDS3 gRNA2 TRV2 Architecture_B with the heat shock treatment, 24 ku70 samples using PDS3 gRNA2 TRV2 Architecture_B with the room temperature treatment, 16 WT samples using PDS3 gRNA2 TRV2 Architecture_A with the heat shock treatment, 57 WT samples using PDS3 gRNA2 TRV2 Architecture_A with the room temperature treatment, 23 WT samples using PDS3 gRNA2 TRV2 Architecture_B with the heat shock treatment, and 57 WT samples using PDS3 gRNA2 TRV2 Architecture_B with the room temperature treatment.

For TRV delivery experiments with PDS3 gRNA12 we analyzed data from 57 WT samples using gRNA12 TRV2 Architecture_B with the room temperature treatment and 34 WT samples using gRNA12 TRV2 Architecture_B with the heat shock treatment.

For TRV delivery experiments with CHLI1 we analyzed data from 47 WT samples using gRNA4 TRV2 Architecture_B with the room temperature treatment and 12 WT samples using gRNA4 TRV2 Architecture_B with the heat shock treatment. We analyzed data from 42 WT samples using gRNA6 TRV2 Architecture_B with the room temperature treatment and 18 WT samples using gRNA6 TRV2 Architecture_B with the heat shock treatment. We analyzed data from 44 WT samples using gRNA9 TRV2 Architecture_B with the room temperature treatment and 11 WT samples using gRNA9 TRV2 Architecture_B with the heat shock treatment.

Authentication

Homozygous Arabidopsis ku70 knock-out line (SALK_123114) was confirmed using primers from the Arabidopsis Biological Resource Center (ABRC). The rdr6 mutant line was confirmed using primers designed in the Jacobsen Lab. Gel electrophoresis confirmed a 616bp deletion in the rdr6 gene body.
