# Supplementary figures and images for: Viral delivery of an RNA-guided genome editor for transgene-free germline editing in Arabidopsis
Source: Nat Plants. 2025 Apr 22;11(5):967–76. doi: 10.1038/s41477-025-01989-9 (PMC12095077; doi:10.1038/s41477-025-01989-9)

Extended Data Fig. 6 Source gel image

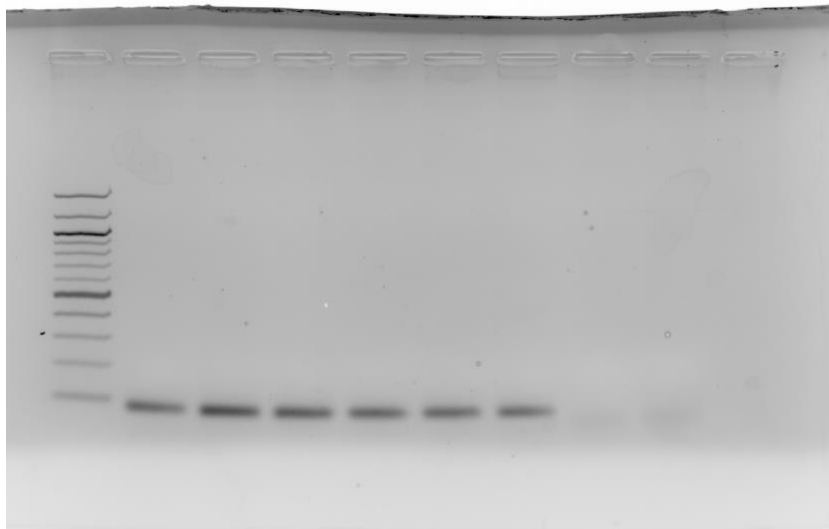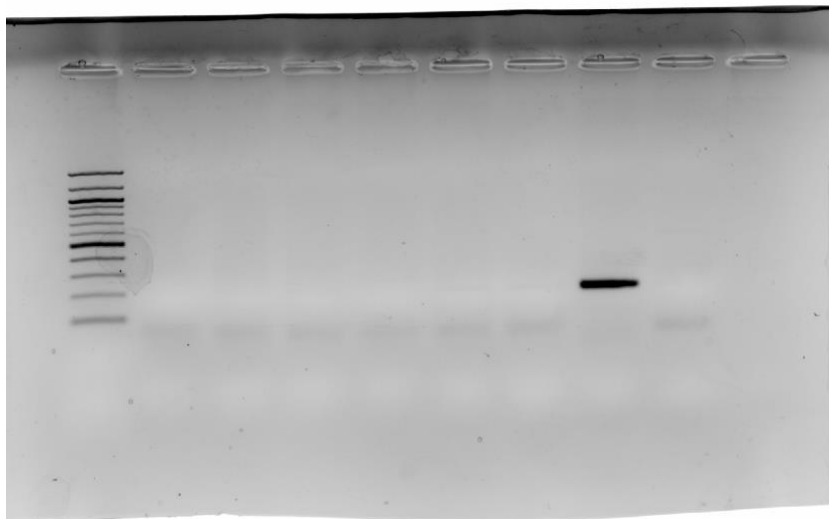

Supplement: Supplementary file 3 — Unprocessed RT–PCR gel. [file 41477_2025_1989_MOESM3_ESM.pdf]
